# Genome-Wide Analysis of *japonica* Rice Performance under Limited Water and Permanent Flooding Conditions

**DOI:** 10.3389/fpls.2017.01862

**Published:** 2017-10-30

**Authors:** Andrea Volante, Francesca Desiderio, Alessandro Tondelli, Rosaria Perrini, Gabriele Orasen, Chiara Biselli, Paolo Riccardi, Alessandra Vattari, Daniela Cavalluzzo, Simona Urso, Manel Ben Hassen, Agostino Fricano, Pietro Piffanelli, Paolo Cozzi, Filippo Biscarini, Gian Attilio Sacchi, Luigi Cattivelli, Giampiero Valè

**Affiliations:** ^1^Research Centre for Cereal and Industrial Crops, Consiglio per la Ricerca in Agricoltura e l’Analisi dell’Economia Agraria, Vercelli, Italy; ^2^Research Centre for Genomics and Bioinformatics, Consiglio per la Ricerca in Agricoltura e l’Analisi dell’Economia Agraria, Fiorenzuola d’Arda, Italy; ^3^Department of Agricultural and Environmental Sciences - Production, Landscape, Agroenergy, Università degli Studi di Milano, Milan, Italy; ^4^Parco Tecnologico Padano, Lodi, Italy; ^5^Istituto di Biologia e Biotecnologia Agraria (CNR), Milan, Italy

**Keywords:** genome-wide association study (GWAS), limited water condition, phenotyping, QTL, rice, tolerance

## Abstract

A rice GWAS panel of 281 accessions of *japonica* rice was phenotypically characterized for 26 traits related to phenology, plant and seed morphology, physiology and yield for 2 years in field conditions under permanent flooding (PF) and limited water (LW). A genome-wide analysis uncovered a total of 160 significant marker-trait associations (MTAs), of which 32 were LW-specific, 59 were PF-specific, and 69 were in common between the two water management systems. LW-specific associations were identified for several agronomic traits including days to maturation, days from flowering to maturation, leaf traits, plant height, panicle and seed traits, hundred grain weight, yield and tillering. Significant MTAs were detected across all the 12 rice chromosomes, while clusters of effects influencing different traits under LW or in both watering conditions were, respectively, observed on chromosomes 4, 8, and 12 and on chromosomes 1, 3, 4, 5, and 8. The analysis of genes annotated in the Nipponbare reference sequence and included in the regions associated to traits related to plant morphology, grain yield, and physiological parameters allowed the identification of genes that were demonstrated to affect the respective traits. Among these, three (*OsOFP2, Dlf1, OsMADS56*) and seven (*SUI1, Sd1, OsCOL4, Nal1, OsphyB, GW5, Ehd1*) candidate genes were, respectively, identified to co-localize with LW-specific associations and associations in common between the two water treatments. For several LW-specific MTAs, or in common among the two treatments, positional co-localizations with previously identified QTLs for rice adaptation to water shortages were observed, a result that further supports the role of the loci identified in this work in conferring adaptation to LW. The most robust associations identified here could represent suitable targets for genomic selection approaches to improve yield-related traits under LW.

## Introduction

Rice is cultivated under both anaerobic (lowland, flooded) and aerobic (upland) conditions, with more than 75% of rice supply coming from 79 million ha of irrigated lowlands (Tuong and Bouman, 2003). Irrigated rice production requires large amounts of water, as 1 kg of rice grain requires 2,500 l of water, so that one third of the World developed freshwater is used to irrigate rice (Bouman, 2009). Water shortages, due to climate change, increasing demand of fresh water from industries and domestic usages, are threatening the sustainability of flooded systems for rice cultivation (Molden et al., 2007; Wassmann et al., 2009). In a slow but steady manner, rice farmers are progressively adopting new cultivation practices involving changes in water management, such as aerobic rice. This crop management, based on periodic drying and re-flooding of rice field, allows 35–50% reduction of water demand (Castañeda et al., 2002; Bouman et al., 2007; Price et al., 2013).

Nevertheless, high-yielding rice varieties bred for lowland cultivation systems are generally subjected to yield loss in aerobic soils (Bouman, 2001; Lafitte et al., 2002; Atlin et al., 2006; Matsuo et al., 2010), so that breeding for traits involved in adaptation to aerobic cultivation needs to be accelerated. Different aspects can contribute to yield penalty in aerobic and/or limited water (LW) conditions, including the exposure of rice plants to mild water stress (Bouman and Tuong, 2001), particularly during the reproductive stage (Atlin et al., 2006; Venuprasad et al., 2007), and the reduced uptake of some nutrients under non-flooded aerobic conditions (Kumar and Ladha, 2011; Vinod and Heuer, 2012).

A rice ideotype adapted for the cultivation under aerobic conditions should possess a combination of morpho-physiological traits such as root distribution in the soil profile, nutrient uptake, and dynamics of tillering and grain filling (Price et al., 2013), which are known to be under complex genetic control. Linkage mapping approaches were used for the identification of QTLs for several traits involved in rice adaptation to aerobic conditions, including drought tolerance, grain yield and root traits (Dixit et al., 2015; Sandhu et al., 2015). A major QTL for grain yield under aerobic cultivation was identified by bulk segregant analysis on chromosome 6, flanked by the SSR markers RM19367 and RM510, and was validated in different environments and genetic backgrounds (Venuprasad et al., 2012). Drought-related QTLs detected across different genetic backgrounds and rice cultivation ecosystems (Kumar et al., 2014), as well as other target regions identified through meta-analysis of drought related QTLs in the Bala × Azucena mapping population (Khowaja et al., 2009) have been reported.

Besides linkage mapping, genome wide association studies (GWAS) represent an additional genetic tool for the identification of rice gene variants that underlie adaptation to aerobic conditions. The emergence of more cost-effective and high-throughput genotyping platforms have allowed the application of GWAS for QTL mapping in plants (Brachi et al., 2011). Since GWAS relies on natural collection of plants, it exploits ancestral recombination events that occurred in population of unrelated individuals and takes into account all major alleles to identify significant marker-trait associations (MTAs), overcoming the constraints of linkage mapping where only a small fraction of all possible alleles is sampled. Therefore, GWAS enables to detect statistically significant loci that explain trait variance using a set of mapped markers, assuming that high-quality phenotypic data, suitable population size and extent of LD, as well as low structured populations are provided (Mackay and Powell, 2007; Zhao et al., 2011).

In the present work, a panel of 281 *japonica* rice accessions genotyped for over 200,000 SNPs (Biscarini et al., 2016) was subjected to association mapping analysis for traits involved in adaptation to LW conditions. For this purpose, the panel was characterized for complex agronomic and physiological traits including heading date, plant height, hundred grain weight (HGW), grain yield expressed as fifty-panicles weight, chlorophyll and flavonoid contents, and nitrogen nutritional status. All the traits were recorded under cultivation in LW and flooded conditions. A substantial number of known and novel QTLs related to adaptation to LW conditions was identified.

## Materials and Methods

### Association Mapping Panel

The accession panel used in this study includes 281 *Oryza sativa* varieties from the Rice Germplasm Collection maintained at CREA-Research Centre for Cereal and Industrial Crops (Vercelli, Italy). This panel is composed of 70 tropical and 211 temperate *japonica* accessions. Most of these accessions (147) were selected in Italy, 32 in United States, 25 in Portugal, 19 in Spain, 10 in Bulgaria, 10 in Argentina, 6 in France, and the remaining were developed elsewhere but considered well adapted to Italian agro-climatic conditions. Detailed information regarding the accessions is reported in Supplementary Table S1.

### Experimental Design

Field trials were set up at the Research Centre for Cereal and Industrial Crops in Vercelli (Italy; coordinates 45°19’204”N, 8°22’25, 35”E – WGS84). The soil in this area is a sandy-type (sand 47.8%, loam 42.8%, clay 9.41%) with pH = 6.36. The entire panel was tested using two different water management systems (permanently flooding – PF, LW). A completely randomized design, with three replicates per watering condition was adopted. Each plot consisted of three 170 cm-long rows 10 cm spaced, each one containing about 60 plants (180 plants/plot). Evaluations were carried out during the 2012 and 2013 growing seasons, following standard agronomic practices for rice growth.

In the PF trial sowing was performed in dry conditions; the field was flooded (10 cm water) when the majority of the cultivars reached the three-leaf stage (typically after 30 days) and kept in this condition throughout the whole growing season until 30 days before harvesting. In the LW trial, sowing was performed as above; six tensiometers (60 cm 2710ARL series, Soilmoisture Equipment Corp.) were evenly installed in the field and watering was applied when the average soil water potential reached values lower than -30 KPa at a depth of 20 cm.

### Phenotypic Evaluation

The 281 rice accessions were phenotyped for the different trait categories listed in **Table 1** and described below.

**Table 1:** List of the measured traits and related units.

Trait category	Trait	Acronym
Phenology	Days to flowering (days)	DF
	Days to maturity (days)	DM
	Days from flowering to maturity (days)	DFM
Plant morphology	Flag leaf length (mm)	FLL
	Flag leaf width (mm)	FLW
	Leaf area (mm^2^)	LA
	Plant height (cm)	PH
	Panicular node height (cm)	PNH
	Panicle length (cm)	PL
Seed morphology	Seed length (mm)	SL
	Naked seed length (mm)	NSL
	Seed width (mm)	SW
	Naked seed width (mm)	NSW
	Seed width/length ratio	SWLR
	Naked seed width/length ratio	NSWLR
Yield	Number of tillers per linear meter	TPM
	Hundred grain weight (g)	HGW
	Hundred naked grain weight (g)	NHGW
	Yield of 50 panicles (g)	PW
Physiology	Chlorophyll content on the abaxial leaf surface	CHL_AB
	Chlorophyll content on the adaxial leaf surface	CHL_AD
	Average chlorophyll content	CHL_AV
	Flavonoid leaf content	FLA
	Nitrogen balance index in the abaxial leaf surface	NBI_AB
	Nitrogen balance index in the adaxial leaf surface	NBI_AD
	Average nitrogen balance index	NBI_AV

#### Phenology Traits

Days to flowering (DF) and days to maturity (DM) were counted starting from the sowing date. Plots were considered as flowered when at least 50% of the plants extruded 1/3 of the panicles, while the maturation date was assumed when at least 50% of panicles showed 2/3 of dried rachis. Days from flowering to maturity (DFM) were calculated as DM – DF.

#### Plant Morphology Traits

Flag leaf length (FLL), flag leaf width (FLW), plant total height (PH), and panicular node height (PNH) were measured on five representative plants for each plot (i.e., excluding outlier sizes). Leaf area (LA) was calculated as FLL^∗^FLW^∗^0.75 while panicle length (PL) as difference between PH and PNH.

#### Seed Morphology Traits

One hundred seeds were randomly chosen from grains obtained from each plot and scanned images of these seeds were analyzed with the software WinSeedlePro V.2011. The morphology descriptors listed in **Table 1** were measured on hulled (SL, SW, SWLR) and naked (NSL, NSW, NSWLR) seeds.

#### Yield Traits

The number of tillers per linear meter (TPM) and yield of 50 representative panicles (PW) were recorded for each plot. The HGW was obtained for each plot using hulled (HGW) and naked (NHGW) seeds.

#### Physiology Traits

Nitrogen Balance Index (NBI), an indicator of the plant nitrogen status (Tremblay et al., 2012), was recorded using the DUALEX 4 Scientific (Dx4) chlorophyll meter developed for the simultaneous estimation of both leaf chlorophyll and epidermal flavonoids to assess the leaf nitrogen content (NBI = CHL/FLA) (Goulas et al., 2004). The sampling was carried out 7–10 days after the flowering date, a period during which the nitrogen status of the plant is stable. In each plot, three measurements were done on the adaxial and the abaxial faces of the panicle leaf of three plants representative of the plot; then, the 30 measurements were averaged to obtain a plot level score of NBI.

### Statistical Analysis of Phenotypic Data

Frequency distributions of phenotypic data were tested for normality using the Shapiro–Wilk function in R environment. Analysis of variance (ANOVA) was performed using the “aov” function in R environment to assess significance of genotypes (G), year (E), genotype × year interaction (G×E) and replicates within each environment (PF and LW). Components of phenotypic variances were estimated by fitting a mixed model by the Restricted Maximum Likelihood method, considering G, E and G×E as random factors. Broad sense heritability (H) was calculated according to Nyquist (1991):

$$\text{H}\;=\;\sigma^{2}G/[\sigma^{2}G\;+\;(\sigma^{2}GE/E)\;+\;(\sigma^{2}e/rE)]$$

where σ^2^*G* is the genetic variance, σ^2^*GE* is the genotype × environment interaction variance, σ^2^*e* is the residual variance, *E* is the number of environments, and *r* the number of replicates. Finally, to find out whether or not traits are correlated to each other, Pearson coefficients were calculated in R using the standard “cor.test” function and the significance of correlations was assessed with the *t*-test implemented in the “cor.test” function.

### Genotypic Data and Genetic Diversity Analysis

The accessions included in the rice panel were genotyped-by-sequencing (GBS) following a pipeline described by Biscarini et al. (2016), except for the number of tags required for the alignment to the Nipponbare reference sequence (1 instead of 5). A set of 246,084 SNPs have been identified, mapped on the Os-Nipponbare-Reference-IRGSP-1.0 pseudomolecule assembly and intersected with the genome annotation (Kawahara et al., 2013) to define the percentage of markers in rice genes.

The original SNP dataset was filtered with the program PLINK^1^ (Purcell et al., 2007) to avoid the biased detections due to rare alleles. Markers with a call rate value lower than 95% and with minimum allele frequency (MAF) lower than 5% were discarded. After filtering for call rate and MAF, a total number of 31,421 SNPs was subsequently used for the GWAS analyses.

### Analysis of Population Structure

To investigate the genetic stratification of the rice panel, three different methodologies based on principal components (PCA), phylogenetic clustering and a Bayesian model-based analysis, were used on a subset of 9,996 random SNP markers (833 markers/chromosome). The PCA was performed using Tassel v5.2.0 (Bradbury et al., 2007) based on the covariance matrix among genotypes. A further description of the population structure was obtained analyzing the phylogeny of rice accessions constructed with the neighbor-joining method and Jukes–Cantor genetic distance using the program MEGA7 (Kumar et al., 2016). Finally, a model-based analysis was performed with Structure, v2.3.4 (Pritchard et al., 2000). The parameters used in this analysis were: presence of admixture, allele frequencies correlated, a burn-in period of 10,000 iterations, followed by 20,000 Monte Carlo Markov Chain (MCMC) replications, K levels from 1 to 10; 5 runs per *K*-value. For the choice of the best number of clusters (K) the Evanno method of ΔK was used, implemented in the free software Structure Harvester (Earl and von Holdt, 2012). Once defined the most probable *K*-value, a final single run was performed using the same parameters listed above, except for the number of burn-in and MCMC iterations (100,000 and 200,000, respectively). Accessions with a minimum membership (i.e., the probability of one individual to belong to a subgroup identified by Structure) of 0.7 were assigned to a subpopulation, while the remaining were considered as admixed. The phylogenetic tree, represented with iTOL^2^ was implemented with the results of the Structure analysis, together with the information relative to the varieties of the panel.

### Linkage Disequilibrium Analysis

The computation of pairwise linkage disequilibrium (LD; *r*^2^) among 5,000 randomly selected markers was performed by the R package “LDcorSV v1.3.1” (Mangin et al., 2012), using the Structure membership matrix as a covariate. The values were averaged in 10 kb windows as in Biscarini et al. (2016). For each distance class, a mean value was obtained from the data of the 12 chromosomes; the resulting values were plotted against physical distance and fitted to a second degree LOESS curve using an R script (Cleveland, 1979; Marroni et al., 2011). A critical value of 0.2 was set as *r*^2^ between unlinked loci. The physical distance corresponding to a LOESS curve value of 0.2 was assumed as LD decay in the rice panel.

### Association Mapping

To unveil the genetic diversity of this rice panel, the number of polymorphic loci, expected heterozygosity (He; Nei, 1978) and the number of transitions and transversions were computed using Arlequin, version 3.5 (Excoffier and Lischer, 2010). These statistics were computed in the rice panel as a whole, in clusters of temperate and tropical *japonica* accessions as well as in the clusters of accessions identified with Structure. The divergence among the populations defined *a priori* according to the subspecies and among clusters identified by Structure, was estimated as *F*_ST_ (Weir and Cockerham, 1984) by the Arlequin software, version 3.5 (Excoffier and Lischer, 2010). The significance of the estimates was obtained through permutation tests, using 1,000 permutations.

For the genome-wide association analysis, the least-square means by year for the traits listed in **Table 1** were calculated. A total number of 31,421 SNPs were used for the analysis. A Mixed Linear Model (MLM) was used for GWAS, with the kinship matrix (K) as a random effect to take into account the population stratification.

Two separate association analyses were performed for the two different watering treatments (PF and LW) with Tassel v5.2.0. The program was run with the following parameters: no compression, genetic and residual variance estimated for each marker (P3D OFF). A *p*-value of the association to the phenotypic traits was calculated for each marker; the significance threshold to declare a marker as associated was set to 0.05 after correction for multiple testing using the false discovery rate (FDR) method according to Benjamini and Hochberg (1995). Manhattan plots and Q–Q plots of each trait were drawn using the R package “qqman” (Turner, unpublished). Single-SNP associations were considered as true positives only when a peak of multiple SNPs was observed at lower –log_10_(*p*-values) in the Manhattan plot (even below the FDR threshold). Clusters of SNPs in full LD showing the same *p*-value in association analysis were considered as a peak region.

The chromosome-wise local LD was calculated with the program Haploview v4.2 (Barrett et al., 2005). LD blocks were defined using the default Haploview settings, i.e., the method by Gabriel et al. (2002), which assumes 0.7 and 0.98 as D’ lower and upper minima for strong LD, respectively. The regions associated to each trait were aligned with the results of the Haploview analysis, in order to detect adjacent associations possibly tagging a single LD block. The regions defined by the peak marker/region positions including 100 kbp upstream and downstream (corresponding to an average LD decay of 0.5 estimated on the LOESS curve described above, as a trade-off between accuracy and power of the analysis) were screened to search for candidate genes underlying each trait. All gene models within these intervals were extracted from the annotation of *Oryza sativa* reference sequence (Os-Nipponbare-Reference-IRGSP-1.0^3^) and reported in Supplementary Table S2 (sheet 1). In order to validate the above results, all annotated gene models included in the selected genomic regions were compared to genes known to be related to the phenotypic traits analyzed and available in the Oryzabase database^4^ (Supplementary Table S3: sheet 2) or available in literature.

## Results

### Phenotypic Variation of Agronomic Traits in Response to LW Conditions

**FIGURE 1:**
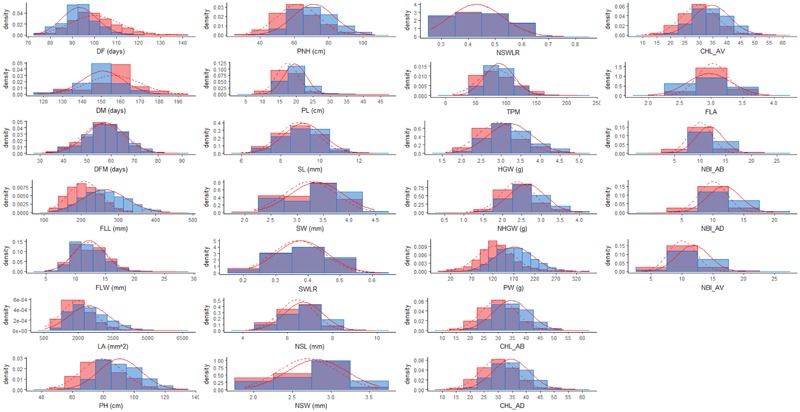
Frequency distribution for all the traits used for the genome-wide association study analyzed in LW (red) and PF (blue) conditions. The normal distributions fitting the LW and PF data were represented with dashed and solid red lines, respectively.

In the present work, a total of 26 traits related to plant phenology, morphology, and physiology (pre-harvest traits) as well as seed morphology and yield (post-harvest traits) were measured on 281 *japonica* rice accessions grown with two water management systems (**Table 1**). The frequency distributions of phenotypic classes indicate that these traits are quantitative and continuous, suggesting a complex genetic control (**Figure 1**). When trait values of rice accessions subjected to the two watering treatments were compared, higher mean values were observed in PF, while for DF, DM, FLW, and FLA no statistically different values were recorded between LW and PF (**Table 2**). However, for average values of DF, DM and DFM, LW resulted in moderately significant delayed flowering, maturation and grain filling period (*P* < 0.05; **Table 2**).

**Table 2:** Summary statistics for the traits evaluated in LW and PF.

	Minimum		Maximum		Mean			*SD*		CV		*H*	
Trait	LW	PF	LW	PF	LW	PF	*P*-value	LW	PF	LW	PF	LW	PF
DF	75.8	75.3	157.33	115.67	100.2	96.17	<0.0001	11.28	8	0.11	0.08	0.87	0.93
DM	129.52	124	186.32	174.17	156.47	150.82	<0.0001	13.33	10.78	0.09	0.07	0.64	0.77
DFM	39.59	40.67	72.67	73.05	56.56	57.62	0.0015	8.52	8.30	0.15	0.14	0.42	0.54
FLL	134.07	142.7	314.17	383.19	208.86	262.61	<0.0001	43.43	58.82	0.21	0.22	0.85	0.86
FLW	8.73	8.1	20.53	22.05	12.47	12.32	0.0668	2.75	2.61	0.22	0.21	0.87	0.88
LA	1036.97	928.86	3547.3	4579.8	1962.49	2448.39	<0.0001	702.42	839.51	0.36	0.34	0.81	0.86
PH	43	48	120.4	135	79.28	90.77	<0.0001	13.6	13.61	0.17	0.15	0.93	0.97
PNH	29.4	35	102	107	61.9	71.1	<0.0001	12.08	12.05	0.2	0.17	0.93	0.97
PL	9.2	10	30	35	17.37	19.6	<0.0001	3.27	3.33	0.19	0.17	0.83	0.83
TPM	33.38	48.84	140.91	144	83.78	88.69	<0.0001	29.88	24.31	0.36	0.27	0.48	0.63
SL	6.74	6.05	11.28	12.17	8.9	9.16	<0.0001	0.99	1.02	0.11	0.11	0.93	0.98
SW	2.35	2.39	4.11	4.45	3.25	3.38	<0.0001	0.49	0.50	0.15	0.15	0.91	0.99
SWLR	0.23	0.23	0.59	0.62	0.37	0.38	0.1837	0.08	0.08	0.22	0.22	0.93	0.99
NSL	4.29	4.25	7.74	8.45	6.42	6.71	<0.0001	0.78	0.85	0.12	0.13	0.91	0.97
NSW	1.96	2.03	3.35	3.59	2.7	2.82	<0.0001	0.38	0.4	0.14	0.14	0.94	0.99
NSWLR	0.27	0.26	0.69	0.74	0.43	0.43	0.7776	0.1	0.10	0.23	0.23	0.94	0.99
HGW	1.97	2.07	4.35	4.75	2.95	3.23	<0.0001	0.54	0.56	0.18	0.17	0.94	0.97
NHGW	1.27	1.63	3.51	3.89	2.39	2.63	<0.0001	0.43	0.47	0.18	0.18	0.94	0.97
PW	22.57	78.06	237.57	315.16	126.74	171.58	<0.0001	43.62	45.36	0.34	0.26	0.77	0.82
CHL_AB	19.64	25.74	47.44	47.76	30.2	34.61	<0.0001	6.35	6.5	0.21	0.19	0.56	0.59
CHL_AD	21.04	26.02	45.49	46.73	30.06	34.57	<0.0001	6.38	6.47	0.21	0.19	0.57	0.59
CHL_AV	20.34	26.19	46.47	47.25	30.1	34.59	<0.0001	6.35	6.46	0.21	0.19	0.57	0.59
FLA	2.65	2.59	3.61	3.40	3.02	2.97	<0.0001	0.24	0.35	0.08	0.12	0.65	0.49
NBI_AB	5.86	8.22	15.93	17.11	10.02	11.8	<0.0001	2.27	2.62	0.23	0.22	0.57	0.54
NBI_AD	6.47	7.41	15.25	16.74	10	11.77	<0.0001	2.25	2.65	0.23	0.23	0.58	0.55
NBI_AV	6.42	8.31	15.59	16.93	10.01	11.8	<0.0001	2.23	2.62	0.22	0.22	0.59	0.55

*SD, standard deviation; CV, coefficient of variation of the panel; H, broad-sense heritability; P-value, significant difference among treatments (LW and PF).*

Differences between the minimum and maximum trait values in PF ranged from 1.3 (for FLA) to 4.9 (for LA) fold changes with the majority of the traits (15 out of 25) showing a fold change higher than 2; whereas for LW, the range of fold changes was from 1.3 (for FLA) to 10.5 (for PW) with 18 traits out of 26 showing a fold change higher than 2. The coefficients of variation (CVs) in LW varied from 8% for flavonoid content (FLA) to 36% for LA and TMP, while in PF CVs ranged between 7 and 34% for DM and LA, respectively (**Table 2**).

**FIGURE 2:**
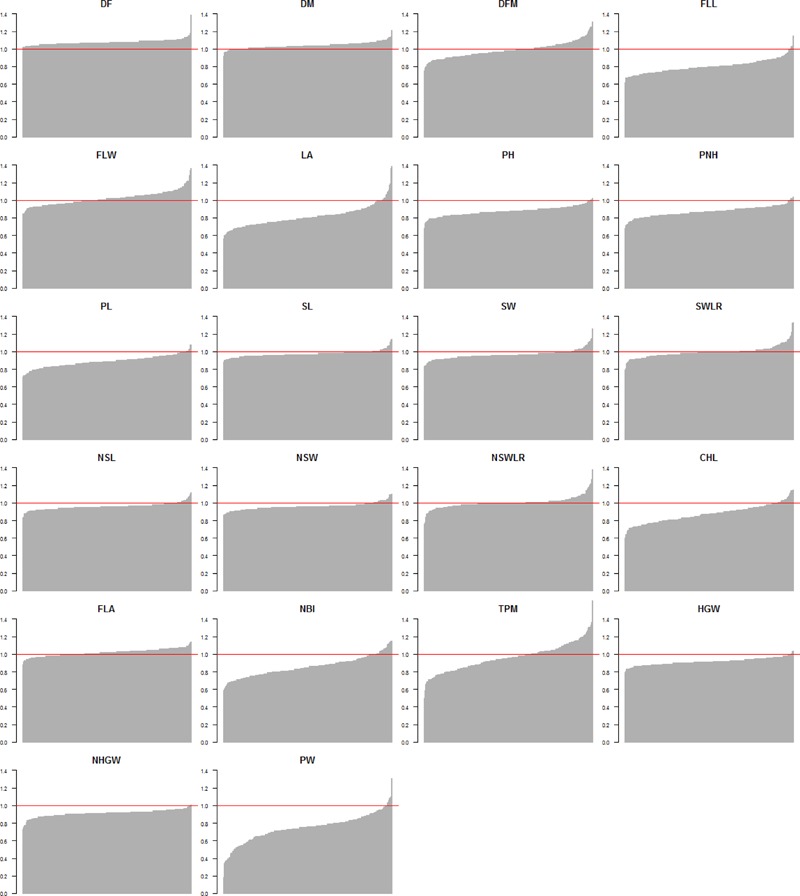
Distribution of the phenotypic value ratio (LW/PF) for all traits used for the genome-wide association study. The red horizontal line represents the 1 value (i.e., accessions showing the same value in both conditions).

The ratios of phenotypic values recorded in the two watering treatments (LW/PF) for all traits and genotypes highlighted that accessions generally performed worst for yield-related traits in LW (**Figure 2** and Supplementary Table S4). However, for some accessions, ratios equal or higher than 1 were observed for PW (eight accessions), TPM (108 accessions) and HGW (seven accessions). Interestingly, considering the eight accessions showing higher LW/PF ratios for PW, only three (Handao11, Campino and Sfera) were included among the 108 accessions with highest TPM ratios, while only Sfera was among the seven accessions with the highest HGW ratios. This indicates that different factors other than tillering and/or grain weight, that could include the seeds per panicle, contributed to the yield performances of these accessions, as assessed through measuring grain production of 50 panicles. Considering phenology-related traits, most of the accessions showed a delayed flowering (all the 281 accessions) and maturation (258 accessions) in LW and 111 genotypes showed a longer flowering to maturation cycle (DFM), even considering that the differences for this last trait were statistically significant for *P* < 0.05 (**Table 2**). In general, the seed width/length ratio, defining the commercial class of each variety, was not influenced by LW. For traits related to plant morphology, 16, 3, and 8 varieties showed higher LA, total plant height (PH) and PL in LW, respectively. Finally, for physiology-related traits, 25 varieties evidenced higher chlorophyll (CHL) and NBI values in LW (**Figure 2** and Supplementary Table S4).

The analysis of variance within each treatment indicates that, in most cases, the effect of the year was highly significant. The genotype effect was always highly significant for all traits in both water management systems (Supplementary Table S4). The broad sense heritability (H) of traits was also reasonably high, varying from 0.42 for DFM to 0.94 for NSW, NSWLR, HGW, and NHGW in LW. In PF the values ranged from 0.49 for FLA to 0.99 for SW, NSW, SWLR, NSWLR, and PNH (**Table 2**). These values suggest that genetic factors greatly contribute to the variance of measured traits.

**FIGURE 3:**
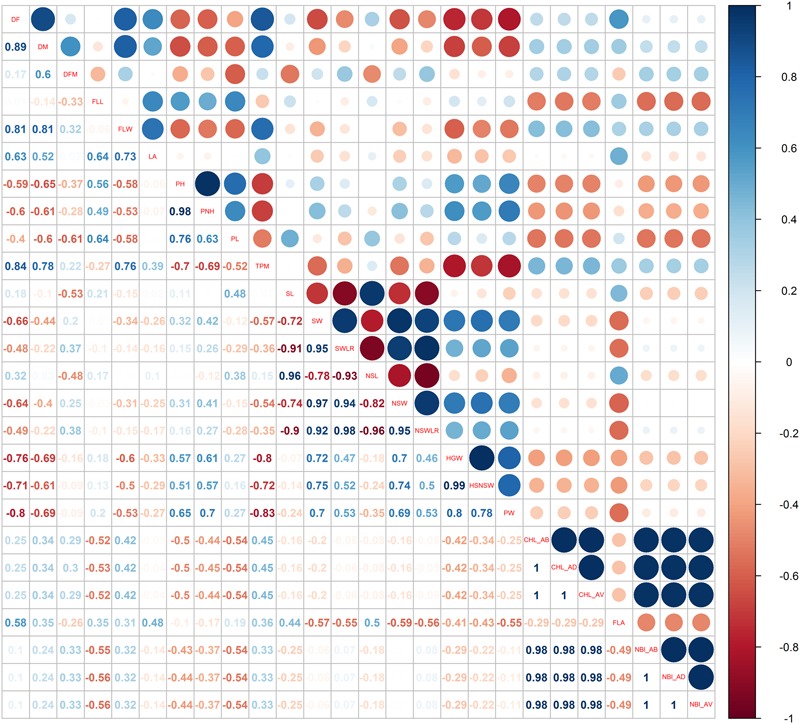
Pearson correlations among the phenotypic traits used for the genome-wide association study recorded in the LW cultivation system.

**FIGURE 4:**
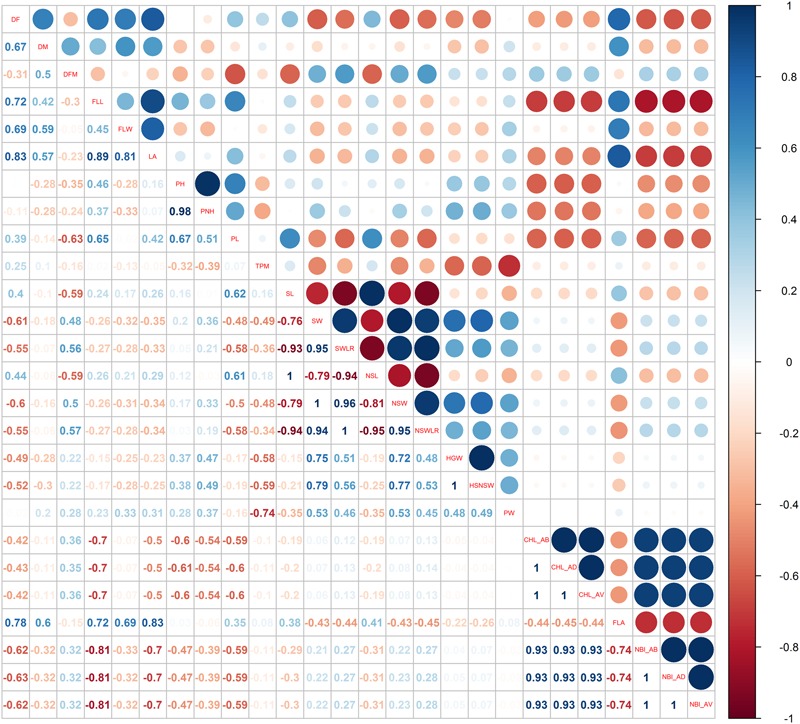
Pearson correlations among the phenotypic traits used for the genome-wide association study recorded in the PF cultivation system.

Pairwise Pearson’s coefficients of correlation among the traits were calculated for each water management system (**Figures 3, 4** and Supplementary Table S2: sheets 3, 4) and, as expected, several traits within each category (phenology, plant and seed morphology, yield and physiology) were highly correlated (*p* < 0.01).

Forty-four correlations specific for the LW condition were identified (highlighted in red in Supplementary Table S2: sheets 3). In particular, under LW, characterized by a delay of flowering date, a positive and highly significant correlation between DF and the number of tillers per square meter (TPM, *r* = 0.84) was observed; on the contrary, DF was negatively correlated with grain and panicle weight (*r* = -0.76 for HGW and *r* = -0.80 for PW). These correlations were not significant under PF conditions (*r* = 0.25, *r* = -0.49, *r* = -0.02 for TPM, HGW and PW, respectively). A negative correlation (*r* = -0.8) was observed between TPM and HGW (**Figure 3**), most likely indicating that under LW, plants producing more tillers had grains of smaller size.

### Genotypic Data and Genetic Analysis

The whole marker set utilized in this study includes 246,084 SNPs, 56.2% of which were mapped on genic regions (coding sequences, genes, exons, mRNAs, 5′ and 3′ UTRs; Supplementary Table S5: sheet 1, total panel). Given an estimated genome size of 373 Mb (Kawahara et al., 2013), the whole genome was covered with an average density of 1.53 kbp/marker (ranging from 1.22 for chromosome 11 to 1.76 for chromosome 5). The subset of marker set used for the association analyses (31,421 SNPs) showed an average density of 11.87 kbp/marker (ranging from 7.03 for chromosome 10 to 18.68 for chromosome 3). In this subset, the percentage of markers mapped on genic regions (51.8% on average) slightly decreased compared to the total set (Supplementary Table S5: sheet 1, GWAS panel).

**FIGURE 5:**
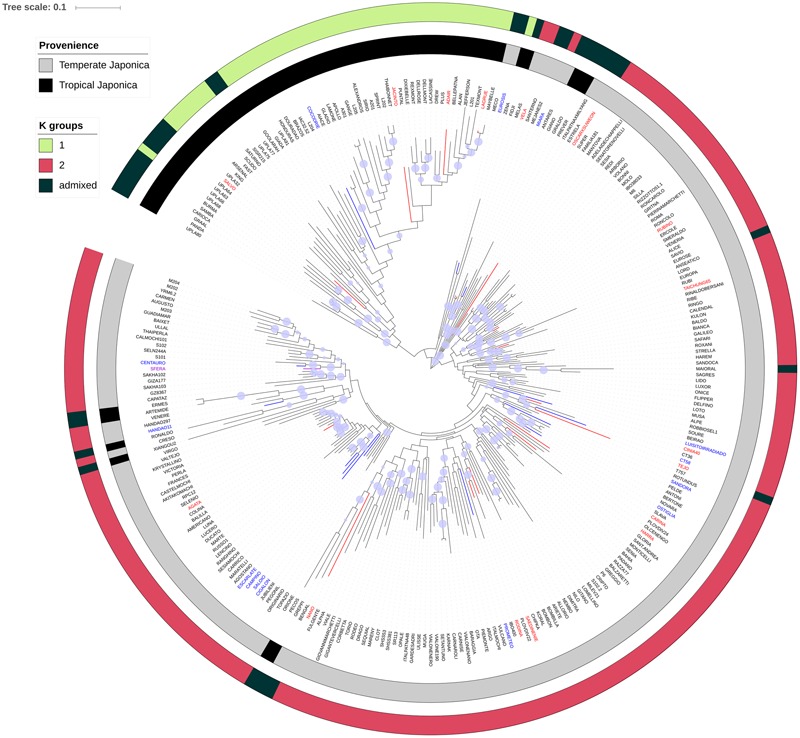
Neighbor-joining tree of the rice panel used in the study. The blue circles on each branch show the results of the bootstrap analysis, when higher than 0.7. The inner gray-and-black coded cycle represents the clustering of the different varieties of the panel according to *O. sativa* classification; the outer cycle (three-color scaled) reports the cluster organization resulting from the STRUCTURE analysis. The rice accessions showing LW/PF values for the PW, HGW, and both traits above the 95 percentile were, respectively, blue, red, and violet highlighted in the neighbor-joining tree.

The analysis of allelic frequencies identified *K* = 2 as the most probable number of Structure clusters in the population, yielding 197 and 49 genotypes assigned to clusters K1 and K2, respectively (with membership > 0.7), and 35 varieties classified as admixed (Supplementary Table S1). PCA (**Supplementary Figure S1**) indicates that the first (PC1) and the second (PC2) coordinates accounted together for 32% of the total variability (28% and 4%, respectively). PC1 clearly separates the sub-populations defined by Structure and corresponding to the temperate and tropical *japonica* subgroups. The admixed accessions clustered in between, reflecting the contribution of both sub-populations in their pedigree. A neighbor-joining clustering of the whole panel, implemented with the classification of the accessions and the membership results from the STRUCTURE analysis, indicates that different clustering methods utilized to unveil the population stratification provided similar results, although a few exceptions were revealed (**Figure 5**). Indeed, 13 varieties classified as tropical *japonica* clustered in the group of temperate accessions and, 10 of them were classified as admixed in the STRUCTURE analysis. The unexpected classification and the admix status of these lines suggest that these rice varieties are the result of breeding programs which exploited accessions from these two rice groups. The position in the neighbor-joining tree of the rice accessions showing LW/PF values above the 95 percentile (**Figure 5**) for the PW and HGW traits did not highlight any obvious clustering, suggesting that alleles conferring adaptation to LW condition in this panel are generally contributed by unrelated accessions. However, considering the LW/PF value for the PW trait, four accessions included in the 95 percentile were clustered, suggesting a common origin of the adaptive alleles in these four accessions, a finding that is supported by the common country of origin (Portugal) for three of them (Campino, Escarlate, and Saloio; Supplementary Table S1).

The genetic diversity calculated for the whole panel was *H* = 0.36, while the same analysis among temperate and tropical *japonica* (*H* = 0.33 and *H* = 0.31, respectively) as well as between the two groups (K1 and K2) identified by the STRUCTURE analysis (*H* = 0.29 in both) resulted in values only slightly smaller (Supplementary Table S5: sheet 2). The genetic divergence between the temperate and tropical *japonica* of the rice panel estimated as *F*_ST_, identified a value of 0.33, whereas considering the two groups identified by the STRUCTURE analysis the *F*_ST_ value was of 0.42. All the comparisons performed were significant at *P* = 0.01.

The mean decay of LD over the physical distance in the whole panel of 281 accessions, calculated as *r*^2^ (**Supplementary Figure S2**) was 911.7 kbp, ranging from 355 kbp for chromosome 11 to 1,295 kbp for chromosome 8 (Supplementary Table S5: sheet 3). When temperate and tropical *japonica* were analyzed separately, the two subpopulations showed comparable LD decay (1,163.3 and 1,150 kbp, respectively; Supplementary Table S5: sheet 3). Overall, these findings suggest that both SNPs and genotype panel considered in this study are useful for carrying out association mapping of complex traits.

### Identification of Marker-Trait Associations Related to Limited Water Conditions

**FIGURE 6:**
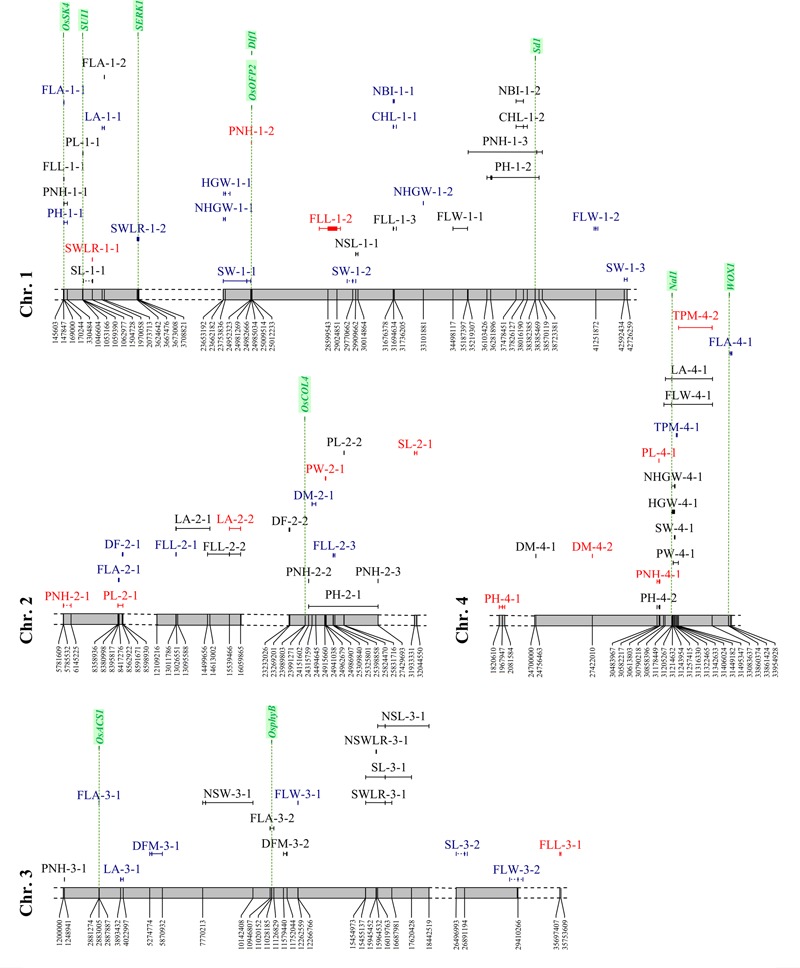
Distribution of the significant associations detected on chromosomes 1 to 4, as indicated in Supplementary Tables S2 and S3 (sheet 1). Acronyms of the traits are as indicated in **Table 1**. Associations LW-specific, PF-specific and common to both watering treatments are represented in red, blue, and black, respectively. Candidate gene locations are shown as green vertical dashed lines with names on top.

**FIGURE 7:**
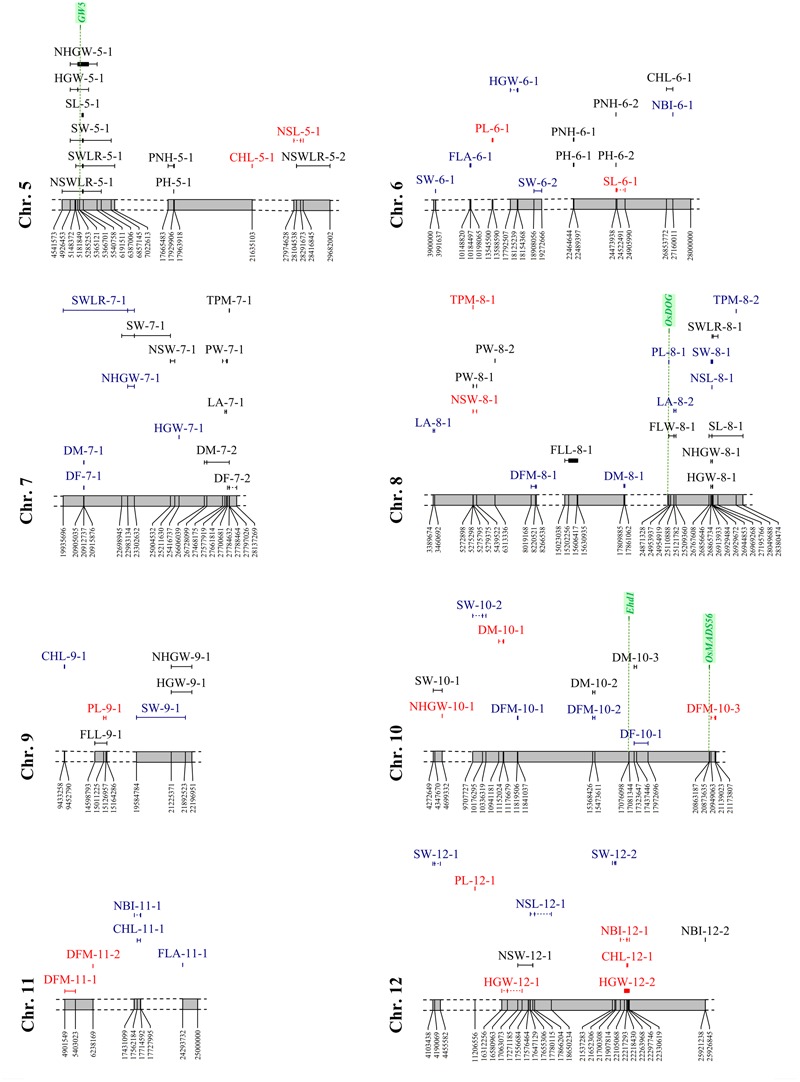
Distribution of the significant associations detected on chromosomes 5 to 12, as indicated in Supplementary Tables S2 and S3 (sheet 1). Acronyms of the traits are as indicated in **Table 1**. Associations LW-specific, PF-specific and common to both watering treatments are represented in red, blue, and black, respectively. Candidate gene locations are shown as green vertical dashed lines with names on top.

A total of 160 significant MTAs were identified for the traits analyzed (**Figures 6, 7, Supplementary Figure S3** and Table S6). The -log_10_(*p*) for these associations ranged between 3.40 and 14.95 (Supplementary Table S6). The lowest number of significant MTAs was detected for physiology-related traits (21), while the plant morphology-related traits showed the highest one (50). Thirty-two MTAs were LW-specific, 59 PF-specific, and the remaining 69 were in common between the two watering treatments. Since 25 loci (single SNPs or sequence intervals) were associated with multiple (2–4) traits, the actual number of significant MTAs was 128. Among them, 105 loci (66%) were defined with two or more markers that co-segregate with QTL peaks (with an average size of sequence intervals of about 654 kbp), while the remaining MTAs were detected by single SNPs. In 23 MTAs, contiguous SNPs in full LD co-segregate with QTL peaks showing exactly the same *q*-value in the association analysis. An analysis of genes underlying the genomic regions where significant MTAs were detected was carried out considering the genes annotated in the Nipponbare reference genome (Supplementary Table S3: sheet 1), in the Oryzabase data (Supplementary Table S3: sheet 2), as well as genes known to explain the phenotypic variance of recorded traits (**Figures 6, 7**). Moreover, in **Supplementary Figure S4** the position on the Nipponbare reference sequence of the peak markers and associated regions of MTAs LW-specific and present in both conditions for which candidate genes were identified, is indicated with respect to the position on the Nipponbare reference sequence of candidate genes. The analysis allowed the identification of positional relationships of several MTAs with genes known to underlie the corresponding traits (Supplementary Table S3: sheet 3). The identified co-positional relationships are indicated below for each trait category.

#### GWAS for Phenology Traits

The analysis yielded 22 significant associations (5 LW-specific, 11 PF-specific, and the remaining present in both conditions), with a percentage of the variance explained (*R*^2^) ranging between 4.9 and 11.4%. A total of 13 associations were detected by more than 1 SNP and 3 of these (DFM-11-1, DFM-3-1 and DFM-3-2) were identified by 11, 12 and 20 markers, respectively. Furthermore, the size of the associated regions ranged from 21,531 to 1,178,593 bp. Three markers were associated with multiple traits: SNP S10_15368426 was associated with DM (both watering treatments) and DFM (PF only), while S7_20912737 and S10_17323647 were associated with both DF and DM (Supplementary Table S6 and **Figures 6, 7**).

Four genes known to underlie flowering time showed positional relationships with regions associated to phenology traits, one of which was specifically detected in LW condition, while the other three genes were in common to LW and PF water managements (**Figures 6, 7, Supplementary Figure S4** and Table S3: sheet 3). This result, together with the phenotypic observation that LW promotes a late flowering, supports the involvement of genes involved in flowering as an adaptive mechanism to the LW condition. The DF-10-1and DM-10-3 (identified in PF and LW, respectively) were located closely to locus *Os10g0463400* representing the *Early heading date 1* (*Ehd1*) gene, which encodes a B-type response regulator promoting flowering by activating the florigen gene *RICE FLOWERING LOCUS T 1* (*RFT1*; Sun et al., 2014). *Os02g0610500*, corresponding to the *OsCOL4* gene, encoding for a CCT domain protein and representing an activator of *Ehd1* (Sun et al., 2014), was located closely to DF-2-2 (common to both, LW and PF). In the region of DFM-3-2 (common to both, LW and PF), *Os03g0309200* is present, which represents the *OsphyB* gene, encoding for a phytochrome and acting as a repressor of *OsCOL4* (Sun et al., 2014). Finally, the sequence interval of DFM-10-3 (LW-specific), encompasses the locus *Os10g0536100* locus which encodes for *OsMADS56*, a repressor of *OsLFL1*, a putative B3 transcription factor whose over-expression decreased *Ehd1* expression and resulted in late flowering (Sun et al., 2014).

#### GWAS for Plant Morphology Traits

A total of 50 MTAs were identified with *R*^2^ values ranging between 4.7 and 20.5% (**Figures 6, 7** and Supplementary Table S6). Among them, two or more markers co-segregate with the peaks of 35 MTAs, while for 5 MTAs (FLL-9-1, LA-4-1, FLW-4-1, PH-1-2, and PNH-1-3) from 24 to 156 SNPs were found to co-segregate with the corresponding QTL peaks. Moreover, the size of the associated regions ranged from 3,587 to 3,504,074 bp. Considering the effect of the water management system, 12 MTAs were LW-specific, 11 were PF-specific, and 27 were in common to both treatments. Seven markers co-segregated with different traits: five SNPs (S2_24151602, S4_30583938, S5_17929906, S6_22464644, and the region S6_24473578-S6_24473604) were in common between PH and PNH, while the region S2_13011786-S2_13026551 was associated with both FLL and LA and S1_170244 was associated with three traits (PH, PNH, and PL).

The analysis of the genes annotated in the Nipponbare reference genome and included in the sequence intervals associated to plant morphology traits revealed some interesting co-localization relationships for six genes (**Figures 6, 7, Supplementary Figure S4** and Table S3: sheet 3). Two of them were localized in LW-specific associations affecting PNH, while three co-localized with MTAs detected in both conditions and affecting PNH, panicle length and leaf traits (FLW and LA). *Os01g0118300*, identified in proximity of the PL-1-1 interval (present in both, LW and PF) corresponds to the *SUI1* (SHORTENED UPPERMOST INTERNODE 1), a gene coding for a putative phosphatidyl serine synthase and representing a negative regulator of the uppermost internode elongation in rice (Zhu et al., 2011). As panicle traits are significantly correlated with internode elongation (Sunohara et al., 2003), the effect of this gene on internode elongation might affect PL.

The sequence interval identified by PNH-1-2, LW-specific, is co-localized with two genes (*Os01g0625900, Os01g0626400*) that were demonstrated to be involved in plant morphology. Rice plants over-expressing *OsOFP2* (*Os01g0625900*) exhibit dwarfism, as this gene modulates NOX and BELL transcription factors, which control vascular development, and showed a reduced expression of the GA biosynthetic enzyme GA 20-oxidase 7 and thus a lower GA content (Schmitz et al., 2015). Also, for *Os01g0626400*, representing the *Dlf1* gene coding for the WRKY11 transcription factor, a positional relationship with the PNH-1-2 region was identified. Mutations on this gene were demonstrated to have pleiotropic effects on flowering time and plant height as mutants exhibited semi-dwarf and late flowering phenotypes (Cai et al., 2014). In particular, *Dlf1* regulates plant height by altering cell size in internodes, supporting a putative implication of this gene in PNH determination. Furthermore, a locus encoding for a GA 20-oxidase (*Os01g0883800*) and corresponding to the “green revolution gene” *Sd1*, whose mutations resulted in a reduced plant height in rice (Monna et al., 2002), was identified in the association detected for PH-1-2 and PNH-1-3, both present in PF and LW (Supplementary Tables S2 (sheet 3), S6).

Finally, the *Os04g0615000* (*Narrow leaf 1: Nal1*) locus was included in the sequence intervals detected by FLW-4-1 and LA-4-1 (both present in LW and PF). Rice mutants in the *Nal1*gene exhibited narrow leaves with a decreased number of longitudinal veins (Qi et al., 2008). Even though *Nal1* function is unknown, it was demonstrated as involved in the regulation of cell division that affect leaf width and plant height by influencing auxin signaling (Bing et al., 2006; Jiang et al., 2015). Moreover, an allele of *Nal1* (SPIKE – SPIKELET NUMBER) increased yield in modern *indica* cultivars as its presence resulted in a higher number of spikelets (Fujita et al., 2013). Combined, these data could support the involvement of *Os04g0615000* in the regulation of FLW and LA in our rice panel.

Also for the PF-specific PL-8-1 interval, the locus *Os08g0504700* (Oryzabase Gene Symbol Synonym *OsDOG*), was identified. The gene encodes for an A20/AN1 zinc-finger which negatively regulates GA homeostasis and thus cell elongation in rice (Liu et al., 2011). It was demonstrated that rice transgenic lines with constitutive expression of *OsDOG* showed dwarf phenotypes, due to deficiency in cell elongation. Moreover, they displayed reduction of GA because of a reduction of the expression of GA3ox2, which allows the production of biologically active GA, and enhanced expression of GA2ox1 and GA2ox3, which mediate conversion of active GA to inactive forms, which lead to dwarf plants and shorter panicles that did not completely emerge from the leaf sheath (Liu et al., 2011).

#### GWAS for Seed Morphology Parameters

The analysis detected 43 MTAs (5 LW-specific, 15 PF-specific and the remaining shared by both conditions) with *R*^2^ values ranging between 6.9 and 19.4% (**Figures 6, 7** and Supplementary Table S6). LW-specific associations were identified for NLS (chromosome 5), NSW (chromosome 8), SL (chromosomes 2 and 6), and SWLR (chromosome 1). Twenty-seven associations were detected by two or more markers with the highest number for NSWLR-5-1, SWLR-5-1, SW-5-1 (266, 269, and 272, respectively) with region sizes ranging between 19,080 and 3,366,936 bp. In general, the number of associations identified for traits measured on hulled grain (SL, SW, SWLR; 7, 15, and 7, respectively) were higher than those for the corresponding traits on de-hulled seeds (NSL, NSW, NSWLR; 5, 6, and 3, respectively), and only four common associations were detected. Markers significantly associated with more traits included the peak markers S3_16378774 and S8_26913933, associated to SL, NSL, and SWLR and the S5_5500521-S5_5538628 region associated with SL, SW, SWLR, and NSWLR. Finally, S1_1504722 was associated with both SL and SWLR and S8_26913933 to SL and NSL. Among the management-specific associations, two (S1_1504722 and S8_26913933) were related to multiple traits. S1_1504722 related to SWLR, was specific for LW and associated to SL in both conditions; while S8_26913933, associated to NSL, was specific to PF and associated to SL in both treatments.

#### GWAS for Yield-Related Traits

Twenty-eight MTAs, with *R*^2^ ranging between 4.4 and 9.0%, were detected for yield-related traits. Eighteen of them were detected by more than one SNP, and the region varied between 8,562 and 1,588,290 bp (Supplementary Table S6). Considering the effect of water management, 7 and 9 associations were LW- and PF-specific, respectively, whereas 12 were shared by both treatments. The number of associations detected for HGW and for the corresponding trait measured on naked grains (NHGW) was comparable (9 vs. 8). Three markers (S1_23753836, S4_31322465, and S8_26929484) were in common between HGW and NHGW and of these, only S8_26929484 was associated to these two traits in both watering treatments (**Figures 6, 7** and Supplementary Table S6). Genes present in the Nipponbare reference sequence where associations for yield-related traits were detected, allowed the identification of three genes (*Os05g0187500, Os04g0615000*, and *Os04g0663600*) which are known to affect yield-related traits (**Figures 6, 7, Supplementary Figure S4** and Table S3: sheet 3). Two of them (*Os05g0187500* and *Os04g0615000*) co-localized with MTAs detected in both conditions and affecting grain weight (HGW and NHGW) and panicle weight (PW). Os05g0187500 encodes for the GW5 (GRAIN WIDTH5) protein and co-localized with HGW-5-1, NHGW-5-1 and other seed traits, present in both LW and PF conditions (**Figure 7, Supplementary Figure S4** and Table S6). This positional relationship is in agreement with the fact that GW5 represents a major QTL underlying rice width and weight and regulates cell division during seed development likely acting in the ubiquitin-proteasome pathway (Weng et al., 2008). Also the *Nal1* gene (Os04g0615000), already described above as associated to leaf-related traits (FLW and LA), is located in the regions associated to panicle (PW-4-1, in both LW and PF) and grain (HGW-4-1, NHGW-4-1, in both LW and PF) weight. Allelic variation at *Nal1* was demonstrated to affect yield acting on the number of spikelets (Fujita et al., 2013). Therefore, this gene could represent a candidate for the HGW-4-1 and PW-4-1 associations.

For the PF-specific TPM-4-1 on chromosome 4, a co-localization with the *WUSCHEL-LIKE HOMEOBOX 1* (WOX1) encoding locus *Os04g0663600* was observed (**Figure 6**). Allelic variations in *WOX1* exhibit correlation with the expression levels of cytokinin regulators and the formation of axillary buds, which in turn deeply affect the tillering capacity (Lu et al., 2015).

#### GWAS for Physiology Parameters

The analysis detected 21 MTAs (3 LW-specific, 13 PF-specific, and 5 in common), with an explained variance (*R*^2^) between 4.5 and 6.5% (Supplementary Table S6). LW-specific associations were identified for CHL (chromosome 5 and 12) and for NBI (chromosome 12, in the same region where the association for CHL was detected), while MTAs under both watering conditions were identified for CHL (chromosome1), FLA (chromosomes 1 and 3) and NBI (chromosomes 1 and 12) (**Figures 6, 7** and Supplementary Table S6). Eleven MTAs were revealed by two or more SNPs and the extent of the regions thus defined ranged between 5,607 and 537,739 bp. Among the specific associations, three were shared by CHL and NBI, which was expected, considering that NBI is derived from CHL (NBI = CHL/FLA) (**Figures 6, 7** and Supplementary Table S6). Candidate genes were identified in the Nipponbare genomic regions where PF-specific MTAs were mapped. *Os03g0152400* encodes a 4-coumarate-CoA ligase-like 1 enzyme (*OsACS1*), a key enzyme of polypropanoids biosynthesis (Falcone Ferreyra et al., 2012). This gene was included in the region associated with flavonoid content identified on chromosome 3 (FLA-3-1, PF-specific). Furthermore, *Os01g0102600*, corresponding to a shikimate kinase (*OsSK4*), showed co-localization with the PF-specific FLA-1-1. Shikimate kinase is a key enzyme of the shikimate pathway, which represents the biosynthetic route for the biosynthesis of aromatic amino acids (phenylalanine, tyrosine, and tryptophan), which building blocks for the production of chorismate and thus of flavonoids (Herrmann, 1995) (Supplementary Table S3: sheet 3).

#### Associations for Multiple Traits Related to Adaptation under LW Condition

In several sequence intervals, significant LW-specific associations related to different traits were identified; these included MTAs on chromosome 4 for PL and PNH (respectively, identified by markers S4_30582194 and S4_30583938), on chromosome 8 for TPM and NSW (respectively, identified by markers S8_5272898 and S8_5275795) and on chromosome 12 for HGW, CHL and NBI (respectively, identified by markers S12_16580963 and S12_22218430; **Figures 6, 7** and Supplementary Tables S2, S6: sheet 1). In addition, also clustering of associations detected under both water management conditions were highlighted; these included MTAs on chromosome 1 for NBI, CHL, PNH and PH (respectively, identified by markers S1_37826127, S1_38457496 and S1_36281896–S1_36346718), on chromosome 3 for NSL, NSWLR, SL and SWLR (respectively, identified by markers S3_16378774, S3_15945566, and S3_16378774), on chromosome 4 for LA, FLW, NHGW, HGW, SW, and PW (respectively, identified by markers S4_31162467, S4_31148130–S4_31153213, S4_31322465, S4_31178449–S4_31243954, S4_31316330, and S4_31257415), on chromosome 5 for NHGW, HGW, SL, SW, SWLR, and NSWLR (respectively, identified by markers S5_5285253, S5_5285253-S5_5789766, and S5_5500521-S5_5538628, this latter in common to SL, SW, SWLR, and NSWLR) and on chromosome 8 for SWLR, SL, NHGW, and HGW (respectively, identified by markers S8_26969268, S8_26913933, and S8_26929484, this latter in common to NHGW and HGW; **Figures 6, 7** and Supplementary Table S6).

Marker-trait associations for yield-related traits were revealed by the marker S10_4699332 that, on chromosome 10, was associated with both SW-10-1 (present in LW and PF) and NHGW-10-1(LW-specific) and marker S7_23302632, that on chromosome 7 was associated with both SW-7-1 (present in LW and PF) and NHGW-7-1 (PF-specific).

## Discussion

### Suitability of the Rice Panel for GWAS Analyses of Rice Adaptation to LW Condition

The average LD decay detected in the rice panel of about 912 kbp was higher than those previously reported (Mather et al., 2007), where values of about 500 kb and 150 kb were indicated for temperate and tropical *japonica* rice, respectively. These discrepancies could be explained considering different factors such as SNP densities and/or kinship among accessions, as previously discussed (Biscarini et al., 2016; Volante et al., 2017). However, higher LD values were also previously observed in rice as ranging from 600 kb up to 2 Mb (Xu et al., 2011; Kumar et al., 2015). These observations suggest that the germplasm panel and markers set used in this work represent excellent resources for investigating rice adaptation to LW condition in *japonica* rice.

Large phenotypic variation for all the traits investigated in the two water management conditions was observed in the panel. Even considering that for most of the genotypes more favorable phenotypic values were recorded under PF condition, for all the traits analyzed performances of several accessions in LW were significantly similar to those obtained under PF. This behavior, together with the reasonably high broad sense heritability values obtained, suggested that the panel was adequate for investigating adaptability to LW conditions in temperate *japonica* rice. Indeed, considering PW as the most important yield-related trait, several accessions were identified as showing values for LW/PF ratios higher or near to 1, suggesting that these accessions should be more adapted to LW management (Supplementary Table S4). Moreover, if the most promising accessions for the LW/PF ratio in the PW trait are considered (e.g., the first 10), it is possible to highlight that these accessions also showed values of the ratios for other yield-, phenology-, morphology-, and physiology-related traits that support their adaptation to LW. As a few examples, the Handao11 line (LW/PF ratio for PW 1.31) showed values near to 1 for NHGW and HGW (0.98 and 0.96, respectively) and values for DF, DM, and DFM approaching 1 (1.07, 1.04, and 1.06, respectively), indicating that flowering parameters in LW are not much affected; Cocodrie (LW/PF ratio for PW 1.09) showed values of 1.17 and 0.95 for HGW and TPM, respectively; Campino, (LW/PF ratio for PW 1.06), also had values of 1.01 for TPM; Sfera (LW/PF ratio for PW 1.03), showed values of 1 and 1.12 for HGW and TPM, respectively; Cigalon, (LW/PF ratio for PW 0.99) recorded ratios of 0.91, 1.29, and 0.95 for HGW, TPM, and PL, respectively; while Escarlate (LW/PF ratio for PW 0.99) showed values of 1.11 and 1.08 for TPM and PL, respectively. Therefore, more traits provide a contribution to the yield values recorded for the accessions ranking at the highest position in the LW system, suggesting that useful adaptive alleles for several traits could be identified in this panel.

### GWAS Analysis for Rice Adaptation to Limited Water Conditions

Physiological mechanisms governing rice adaptation to LW conditions have recently been extensively reviewed (Price et al., 2013) and involve complex mechanisms which include the adaptability to different levels of oxygen availability, that decreases during flooding, leading to an increase of shoot ACC (1-aminocyclopropane-1-carboxylic acid, the ethylene precursor), and decrease of shoot ABA (abscisic acid) and cytokinin. Conversely, aerobic and drying increase shoot ABA (and possibly ACC) and decrease shoot cytokinin. Conditions of alternate aerobic and anaerobic conditions are also expected to alter macro and micro-nutrients availability and uptake, such as phosphorus, which is more available in flooded and anoxic soils, and the redox chemistry of soils. Root-related traits are therefore expected to play important roles in adaptation to changing levels of water availability since their placement with respect to the timing and intensity of fluctuations of water availability and redox potential will affect nutrient access and the signaling between root and shoot (Price et al., 2013). Limitation in water availability also affect the aerial part of the plant since it is recognized that floral fertility in rice is extremely sensitive to water stress (Richards et al., 2010).

Considering the large number of parameters involved in adaptation to LW conditions, a remarkable number of 26 traits were monitored in 281 rice accessions grown in LW and PF in this study. Despite the high extent of LD, which is known to decrease the resolution of GWA studies, this approach allowed the identification of 32 LW-specific MTAs and 69 associations which were in common between the two water systems. Of the 32 LW-specific associations, 5 were for phenology-, 3 for physiological-, 12 for plant morphology-, 4 for seed morphology-, and 7 for yield related-traits. LW-specific MTAs are expected to derive from alleles present only in accessions that are more or less (depending from the allelic contribution to the trait) adapted to this water condition. Accumulation of these alleles conferring small fractions of improved phenotypic values for a given trait, as suggested by the estimated *R*^2^ values (Supplementary Table S6), is expected to provide rice lines with improved adaptation to LW conditions. A similar output could be expected when loci showing associations detected in both the conditions (LW and PF) are pyramided in an improved rice line. In this study, for yield-related traits, seven significant associations were identified in the LW only and 12 significant associations have been highlighted as significant under both water management systems.

Using grain yield under reproductive-stage drought as a selection criterion, a number of large-effect QTLs for grain yield under reproductive-stage drought for both upland and lowland conditions have been identified and recently reported (Kumar et al., 2014). Positional relationships among these drought QTLs and MTAs detected in this work under LW and common to the two watering conditions are described in the following sections for each trait category. Moreover, it was also carried out a comparison of MTAs detected in the present work with the sequence interval of drought-related QTLs identified through a meta-QTLs analysis in the Bala × Azucena rice mapping population (Khowaja et al., 2009) and with MTAs identified in a previous study conducted with a GWAS panel related to the one used in this work (Biscarini et al., 2016). Finally, MTAs for which putative candidate genes were identified and described in the results section are here re-called (**Figures 6, 7** and **Supplementary Figure S4**) with the final aim of establishing multiple relationships for the MTAs identified and providing a list of robust MTAs for adaptation to LW conditions.

#### Yield-Related Traits

Among the LW-specific associations, PW-2-1 (from 24,941,038 to 24,962,679 bp), NHGW-10-1 (peak marker position at 4,699,332 bp) and HGW-12-1 (peak marker position at 16,580,963 bp; Supplementary Table S6) showed co-positional relationships with the physical regions highlighted by qDTY2.2 (2,020,512–25,865,568 bp), qDTY10.1 (5,352,766–18,655,769 bp), and qDTY12.1 (14,106,460–18,155,593 bp), respectively (Kumar et al., 2014). Moreover, the intervals defined by the LW-specific associations HGW-12-1 and HGW-12-2 (from 16,580,963 to 22,297,746 bp; Supplementary Table S6) overlapped with a 20 Mb region defined by the markers RM247 and RG543 markers (from 3,185,384 to 23,775,332 bp) as related to drought avoidance by the meta-analysis (Khowaja et al., 2009).

Finally, also the MTAs HGW-5-1, NHGW-5-1 and other seed traits, showing a positional relationship with the *GW5* candidate gene, and PW-4-1, which co-localize with the *Nal1* candidate gene (described in the Results section), are present in both LW and PF conditions and could represent robust associations (Supplementary Table S6).

#### Phenology-Related Traits

For this category of MTAs, it was observed that DFM-3-2 (present in LW and PF, peak marker at 11,731,550 bp) overlapped with sequence intervals related to drought avoidance on chromosome 3 (Khowaja et al., 2009), identified by the markers RZ474 and R1618 (from 25,128,239 to 30,720,415 bp). DFM-3-2 was also found to map close to *OsphyB* candidate gene (Results section), further reinforcing this association. Similarly, DM-4-2 (LW-specific, peak marker at 27,422,010 bp) overlapped with a sequence interval on chromosome 4 delimited with markers C513 and RM349 (from 22,349,484 to 32,499,619 bp), which controls drought avoidance.

Additional robust loci include DM-10-3 (LW-specific) closely located to the *Ehd1* gene, DF-2-2 (common to both, LW and PF), which lies in the region of the *OsCOL4* gene, DFM-3-2 (common to both, LW and PF) and DFM-10-3 (LW-specific), which is in the region of the *OsMADS56* candidate.

#### Plant Morphology-Related Traits

Among the several associations detected for leaf morphology, FLW-4-1 (present in LW and PF conditions, peak marker at 31,148,130 bp) overlapped with a 775.7 kb interval identified by Biscarini et al. (2016) with the marker S4_31080152, as associated to the same trait and co-localized with the *Nal1* gene. Furthermore, the association FLL-1-2 (LW-specific, peak marker at 28,599,543 bp) was included in a LD block spanning the interval 28,186,751–29,184,329 bp, containing the S1_28597986 marker identified by Biscarini et al. (2016) as associated to FLL. Finally, the associations FLW-3-1 (LW-specific, peak marker at 35,697,407 bp) and FLL-9-1 (present in LW and PF conditions, peak marker at 14,598,793 bp), were previously detected by Biscarini et al. (2016) as associated to the same trait.

The PH-related associations PH-6-1, PH-6-2, PNH-6-1, and PNH-6-2, all present in LW and PF conditions, are in a large LD block (22,367,525–22,678,707 bp) which included the marker S6_22330734 associated to the same trait by Biscarini et al. (2016). In this region the *D35* and *HDA702* genes are localized. Allelic variation in the first one demonstrated to be implicated in the GA biosynthetic pathway, therefore influencing GA levels which in turn affects plant height (Itoh et al., 2004; Matusmoto et al., 2016); instead, *HDA702* encodes for a histone deacetylase involved in plant growth and architecture through an epigenetic repression of *OsNAC6* (Chung et al., 2009). The associations PNH-1-3 (present in LW and PF, peak marker at 38,457,496 bp), PNH-3-1 (present in LW and PF, peak marker at 1,248,941 bp), and PL-12-1 (LW-specific, peak marker at 11,206,556 bp) overlapped, respectively, with three genomic regions on chromosome 1 (around the marker B1065E10: from 38,245,917 to 40,367,906 bp), chromosome 3 (flanked by markers RM3894 and R1618 from 1,116,926 to 30,720,415 bp), and chromosome 12 (flanked by markers RM247and RG543: from 3,185,384 to 23,775,332 bp) identified as related to drought avoidance through meta QTL analysis (Khowaja et al., 2009).

Additional robust loci, are PL-1-1 (present in both LW and PF), which is located in proximity of the *SUI1* candidate gene, the genomic interval identified by PNH-1-2 (LW-specific) which co-localized with the *OsOFP2* and *Dlf1* genes, PH-1-2 and PNH-1-3 (present in both PF and LW) which includes the “green revolution gene” *Sd1* and LA-4-1 (present in both LW and PF), which co-localizes with the *Nal1* gene.

#### Seed Morphology-Related Traits

The interval 5,500,521–5,538,628 bp on chromosome 5 co-segregates with the peak of SW-5-1, SWLR-5-1, and NSWLR-5-1 (present in both LW and PF; Supplementary Table S6). It includes a 254 kbp region previously identified by Biscarini et al. (2016) associated to the same traits, with the S5_5401194 marker as a peak. This region contains the candidate major QTL *GW5*. Furthermore, overlapping with drought avoidance-related regions (Khowaja et al., 2009) were identified for: (i) LW-specific association SL-2-1 (peak marker at 31,933,331 bp) with the chromosome 2 region defined by markers a18438 and C601(from 22,596,168 to 30,270,847 bp); (ii) five MTAs on chromosome 3 (present in LW and PF) as related to seed morphology (NSW-3-1, NSWLR3-1, SL-3-1, NSL-3-1, and SWLR-3-1, with peak markers ranging from 7,907,626 to 16,378,774 bp) which overlapped with a 25 Mb interval defined by markers RG191 and R1618 (from 5,729,669 to 30,720,415 bp).

For MTAs of physiology-related traits LW-specific, or identified in both watering conditions, no positional relationships were recorded with previously identified QTLs or candidate genes. Therefore, the three LW-specific and five MTAs in common among LW and PF cannot be supported by additional data.

Robust loci identified in this work, which should confer advantage under LW could represent suitable targets for Genomic Selection approaches to improve yield under LW conditions. As example, the GWAS panel used in this study has recently been used as a reference population in Genomic Selection to estimate the average genomic prediction accuracies within the reference population itself (cross validation) and genomic prediction of lines-progenies of bi-parental crosses involving accessions belonging to the reference population (across generations) for complex traits investigated also in the present work, namely flowering date, nitrogen balance index, and yield-related traits. In the Genomic Selection study, we observed that the use of phenotypic and genotypic data from the reference population to train the prediction model allowed the prediction of the performances, in both the approaches (cross validation and across generations) with accuracies superior to 0.5, even for complex traits such as grain yield, when the parameters that affect the accuracy are optimized (Ben Hassen et al., in revision).

## Conclusion

Results obtained in the present work indicate that most of the 281 rice temperate and tropical accessions of our panel are penalized for several agronomically relevant traits when grown under LW. However, performances of several accessions in LW were similar to those obtained under PF, suggesting that genetic variability for adaptation to LW conditions is present in our *japonica* rice panel. The GWAS analysis provided a large number of significant associations, an expected result considering the high number of phenotypic traits investigated, and, among them, 32 were LW-specific, while 69 were in common between LW and PF. Genomic regions where clustering of multiple traits affecting performances under LW and both water management conditions were identified and could be hot-spots for adaptation to LW conditions. The robustness of several of these effects was assessed through the identification of genes whose allelic variation could affect the phenotypic response to LW and through verification of positional relationships with QTLs previously identified as involved in rice adaptation to drought stress or reduced water availability. Since the GWAS panel used in this study was successfully used in a recent Genomic Selection approach, involving both cross validation and genomic prediction across generations (Ben Hassen et al., in revision); it is therefore expected that the accumulation of alleles here identified as conferring improved phenotypic values under LW or both cumulated (LW and PF) conditions through Genomic Selection should allow recovery of *japonica* rice lines with improved adaptation to LW conditions.

## Author Contributions

AnV, FD, AT, and CB carried out the data analyses and wrote the manuscript. FD, CB, RP, GO, PR, DC, SU, and MBH participated in field and post-harvest evaluations of phenotypic traits. AnV, FD, AF, PC, and FB performed the data analysis and revised the paper. GS, PP, LC, and GV designed the study and revised the paper. All authors have read and approved the final manuscript.

## Conflict of Interest Statement

The authors declare that the research was conducted in the absence of any commercial or financial relationships that could be construed as a potential conflict of interest.

## Footnotes

1 http://zzz.bwh.harvard.edu/plink/

2 http://itol.embl.de/

3 http://rapdb.dna.affrc.go.jp/download/irgsp1.html

4 https://shigen.nig.ac.jp/rice/oryzabase/

## Supplementary Material

FIGURE S1 Principal coordinate analysis of the rice panel used in the study; point colors correspond to a different taxonomic group defined in literature **(Top)** or to a different cluster obtained in the STRUCTURE analysis at *K* = 2 **(Bottom)**.

FIGURE S2 Average LD (*r*^2^, blue curve) as a function of marker physical distance in the panel of the 281 accessions. The red curve represents the second grade LOESS curve which approximates the *r*^2^ point distribution. The horizontal red line shows the critical *r*^2^ level between linked markers.

FIGURE S3 Manhattan plots and QQ plots of the significant associations detected for each phenotypic trait.

FIGURE S4 Position (bp) on the Nipponbare genome of the peak markers (small black boxes) and associated regions for MTAs LW-specific (in red) and in common to the two watering managements (in black) with respect to the position (bp) on the Nipponbare genome of the candidate genes identified for the indicated MTAs.
