# Supplementary figures and images for: Genome-Wide Analysis of japonica Rice Performance under Limited Water and Permanent Flooding Conditions
Source: Front Plant Sci. 2017 Oct 30;8:1862. doi: 10.3389/fpls.2017.01862 (PMC5670151; doi:10.3389/fpls.2017.01862)

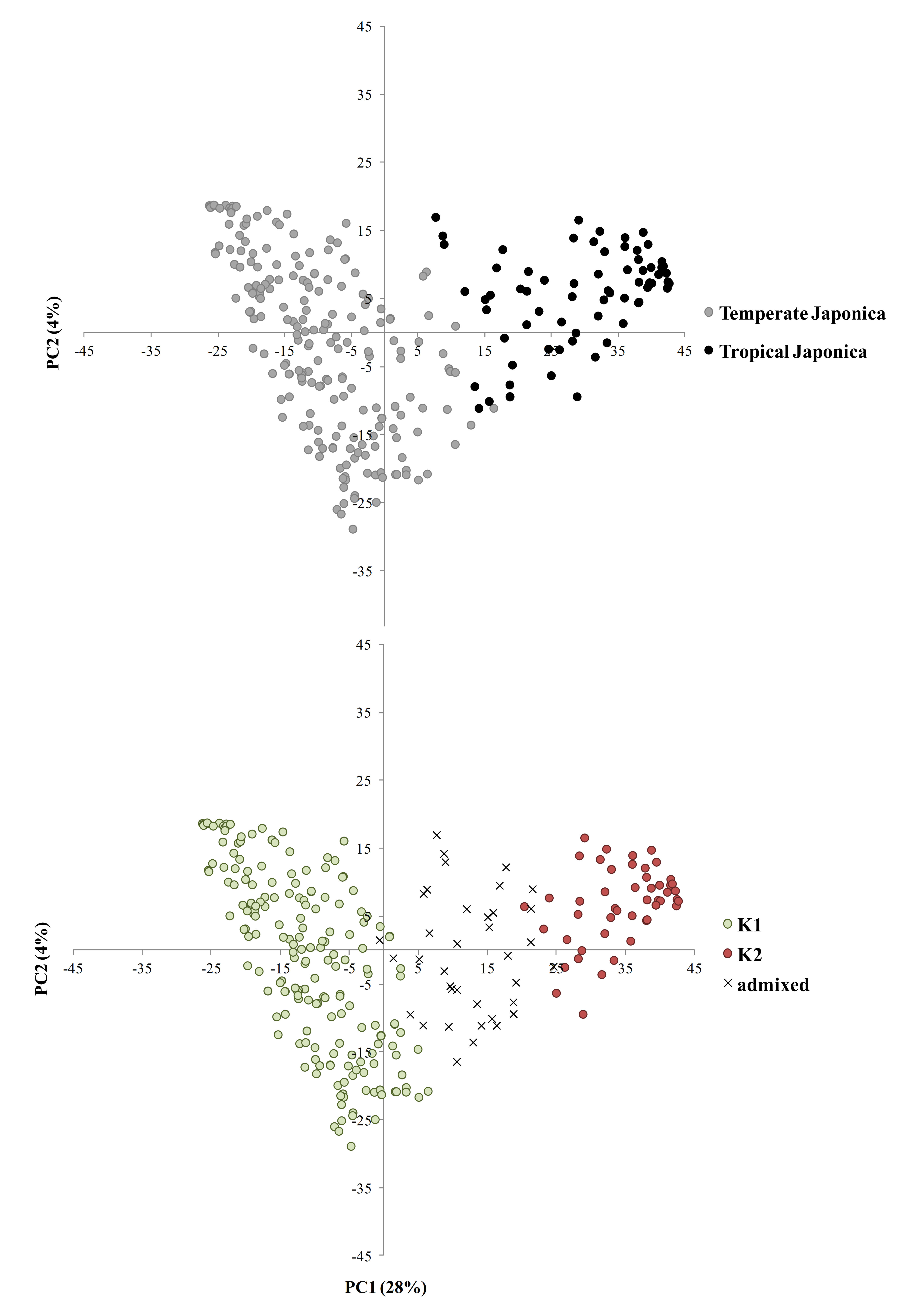

Supplement: FIGURE S1 — Principal coordinate analysis of the rice panel used in the study; point colors correspond to a different taxonomic group defined in literature (Top) or to a different cluster obtained in the STRUCTURE analysis at K = 2 (Bottom). [file Image_1.TIF]

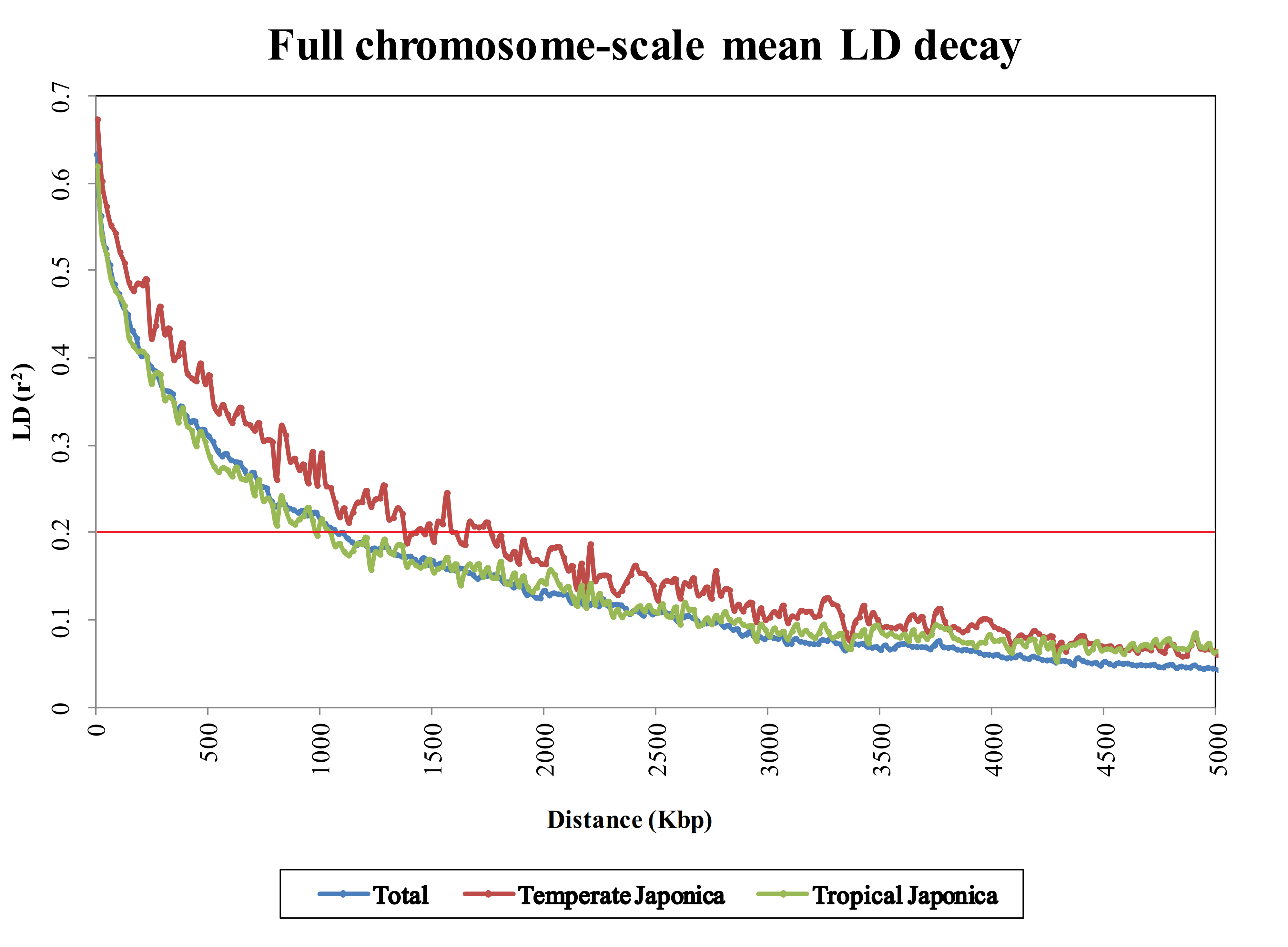

Supplement: FIGURE S2 — Average LD (r2, blue curve) as a function of marker physical distance in the panel of the 281 accessions. The red curve represents the second grade LOESS curve which approximates the r2 point distribution. The horizontal red line shows the critical r2 level between linked markers. [file Image_2.TIF]

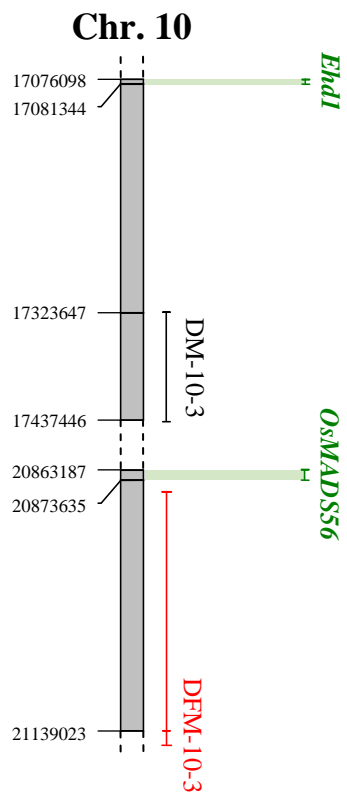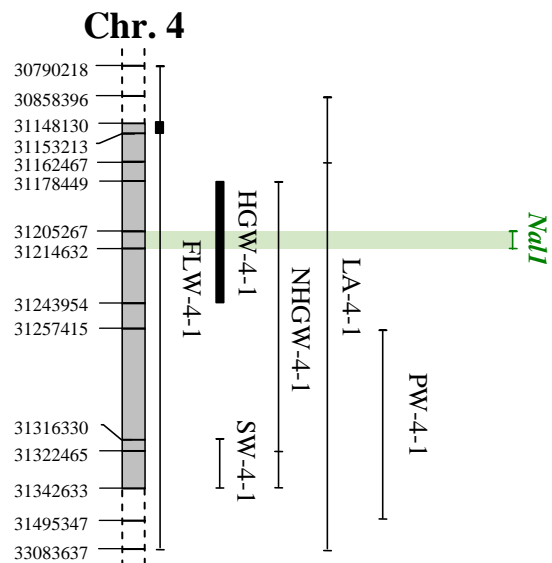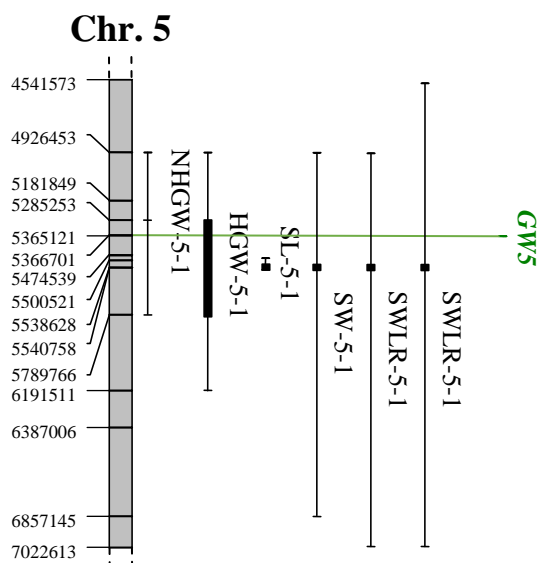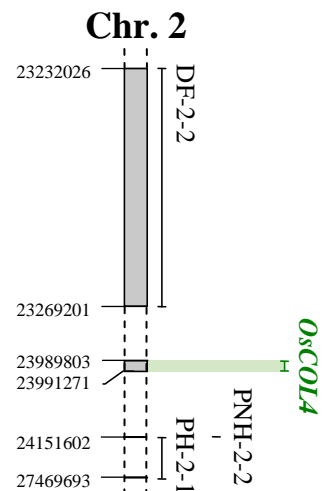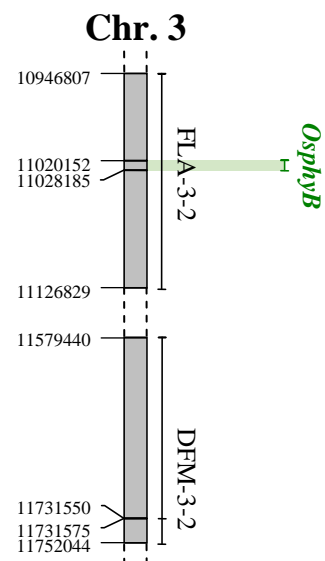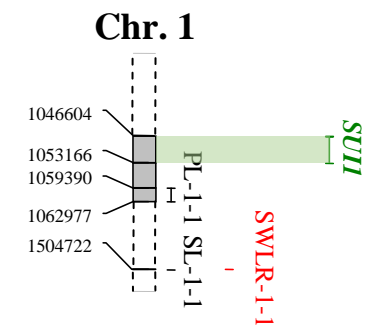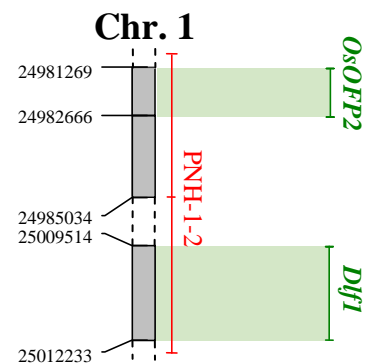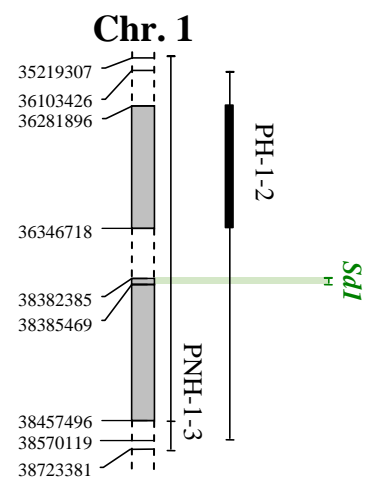

Supplement: FIGURE S4 — Position (bp) on the Nipponbare genome of the peak markers (small black boxes) and associated regions for MTAs LW-specific (in red) and in common to the two watering managements (in black) with respect to the position (bp) on the Nipponbare genome of the candidate genes identified for the indicated MTAs. [file Image_4.PDF]
