# Supplementary material for: Genome-Wide Analysis of japonica Rice Performance under Limited Water and Permanent Flooding Conditions
Source: Front Plant Sci. 2017 Oct 30;8:1862. doi: 10.3389/fpls.2017.01862 (PMC5670151; doi:10.3389/fpls.2017.01862)
Supplement: FIGURE S3 — Manhattan plots and QQ plots of the significant associations detected for each phenotypic trait. [file Image_3.pdf]

PHENOLOGY TRAITS

DF_LW

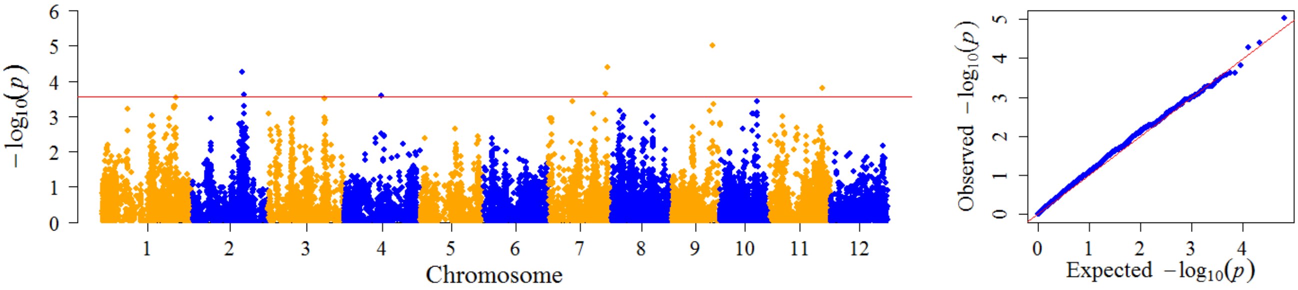

DF_PF

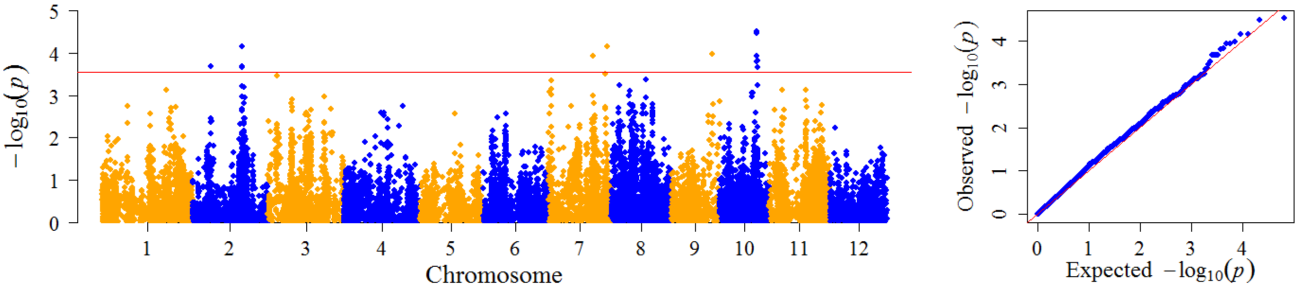

DM_LW

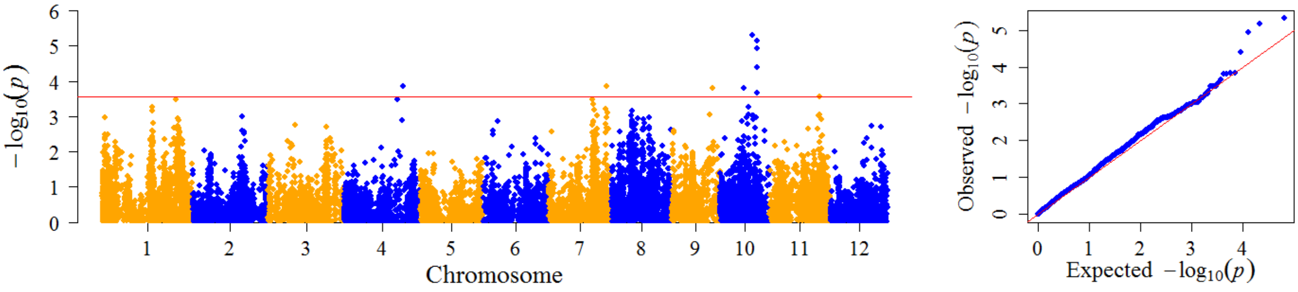

DM_PF

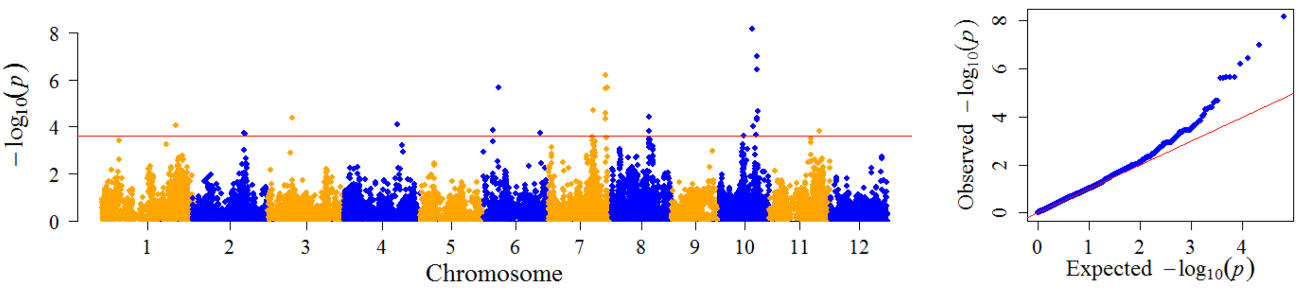

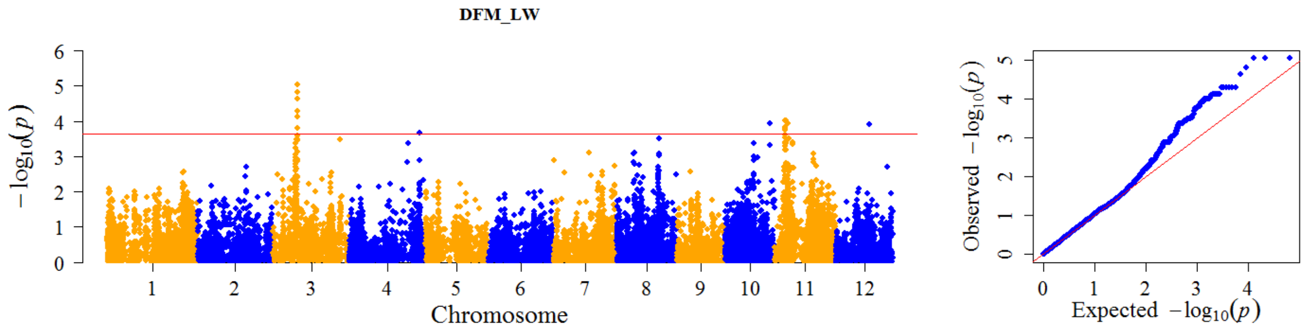
DFM_LW
$-\log_{10}(p)$
Chromosome
Observed $-\log_{10}(p)$
Expected $-\log_{10}(p)$

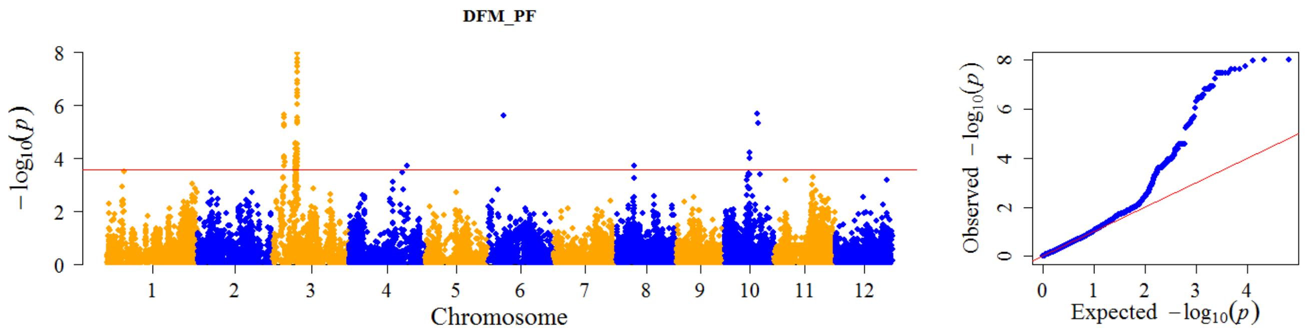
DFM_PF
$-\log_{10}(p)$
Chromosome
Observed $-\log_{10}(p)$
Expected $-\log_{10}(p)$

PLANT MORPHOLOGY TRAITS

LA_LW

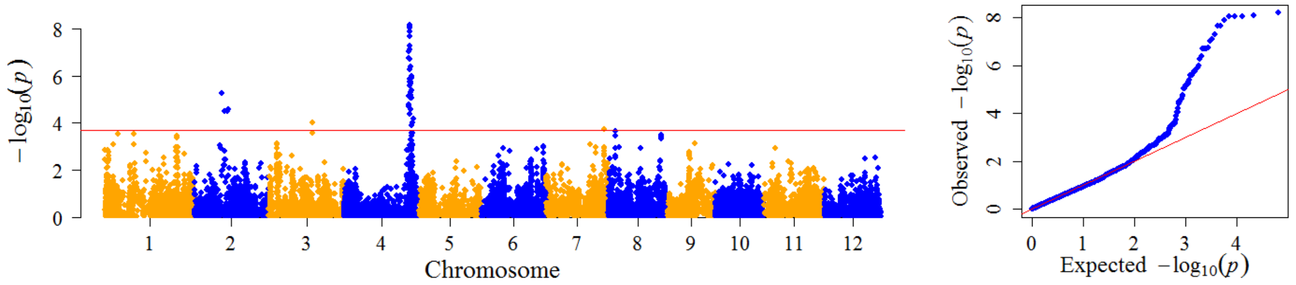

LA_PF

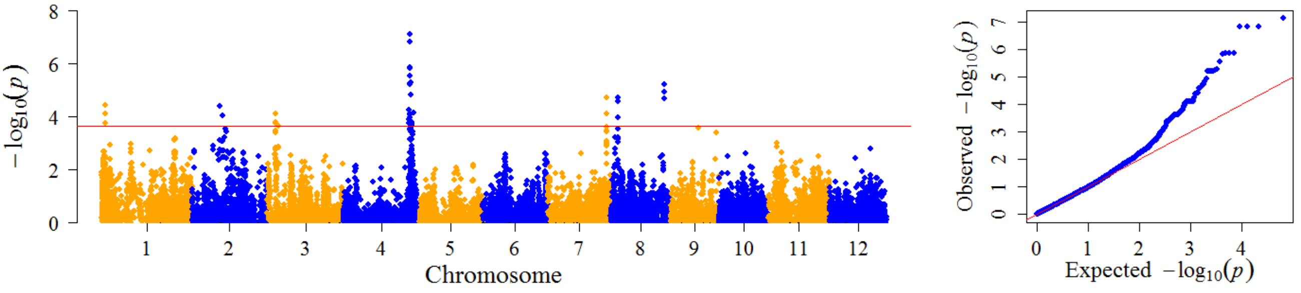

FLL_LW

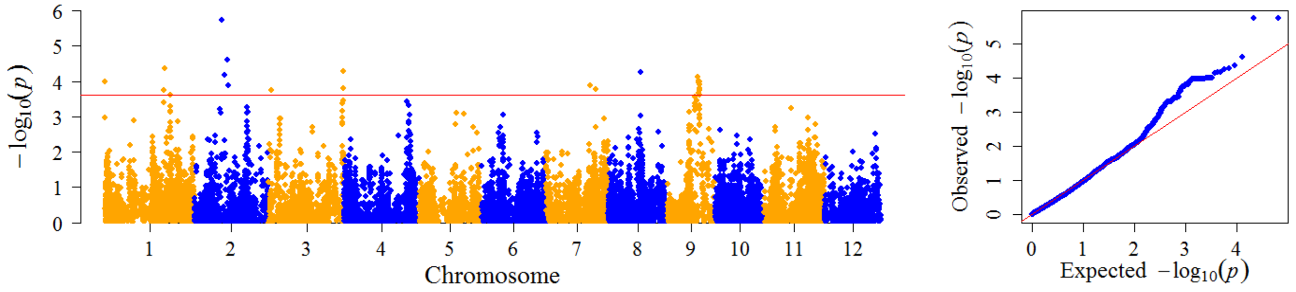

FLL_PF

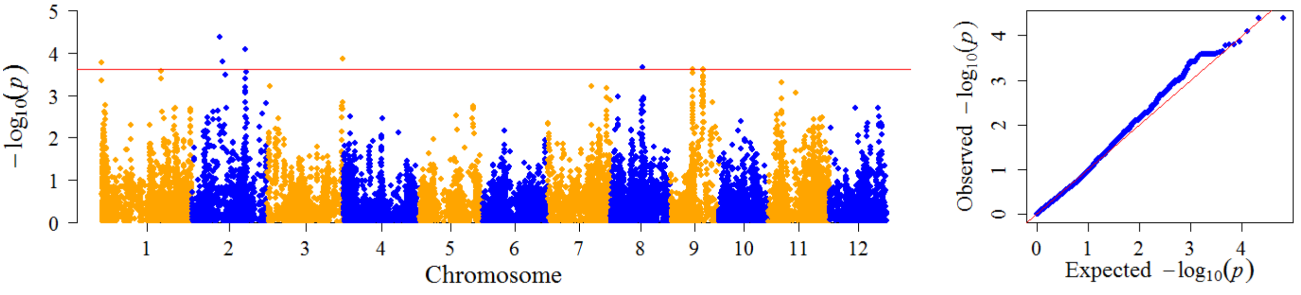

FLW_LW

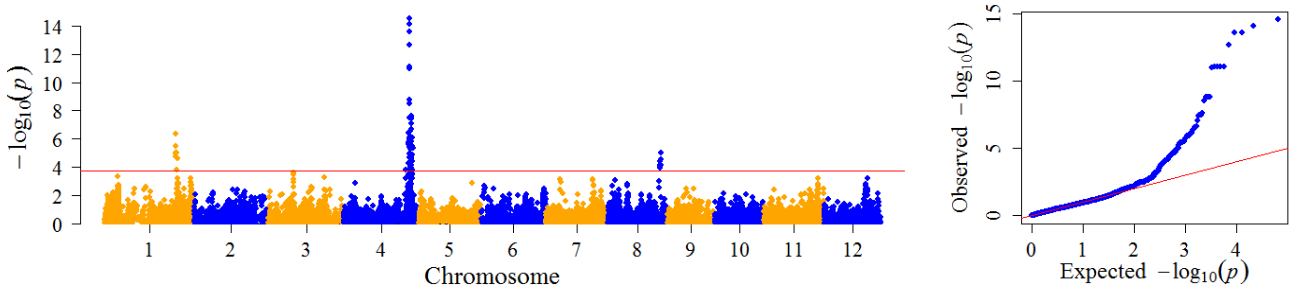

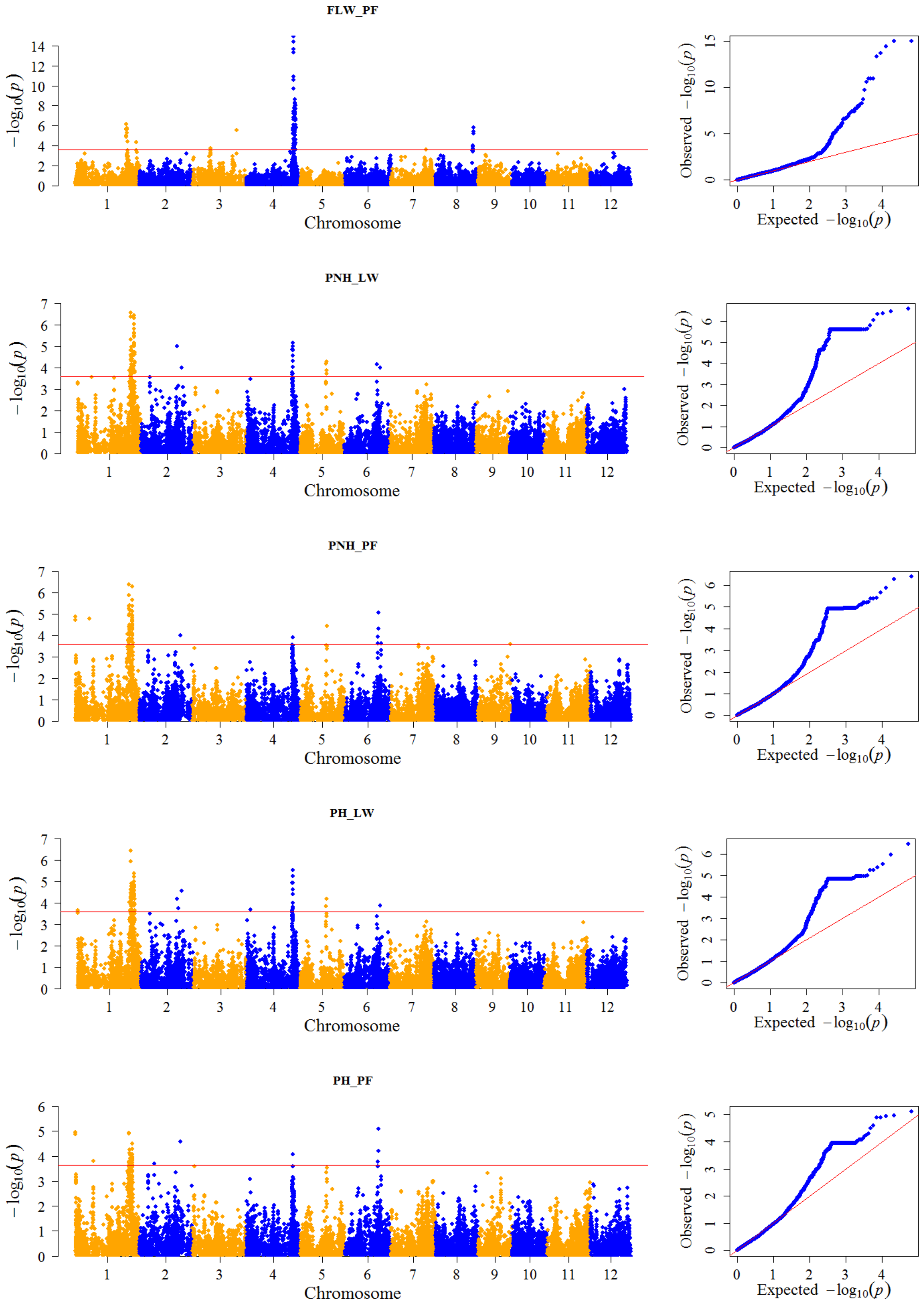
FLW_PF
PNH_LW
PNH_PF
PH_LW
PH_PF
$-\log_{10}(p)$
Chromosome
Observed $-\log_{10}(p)$
Expected $-\log_{10}(p)$

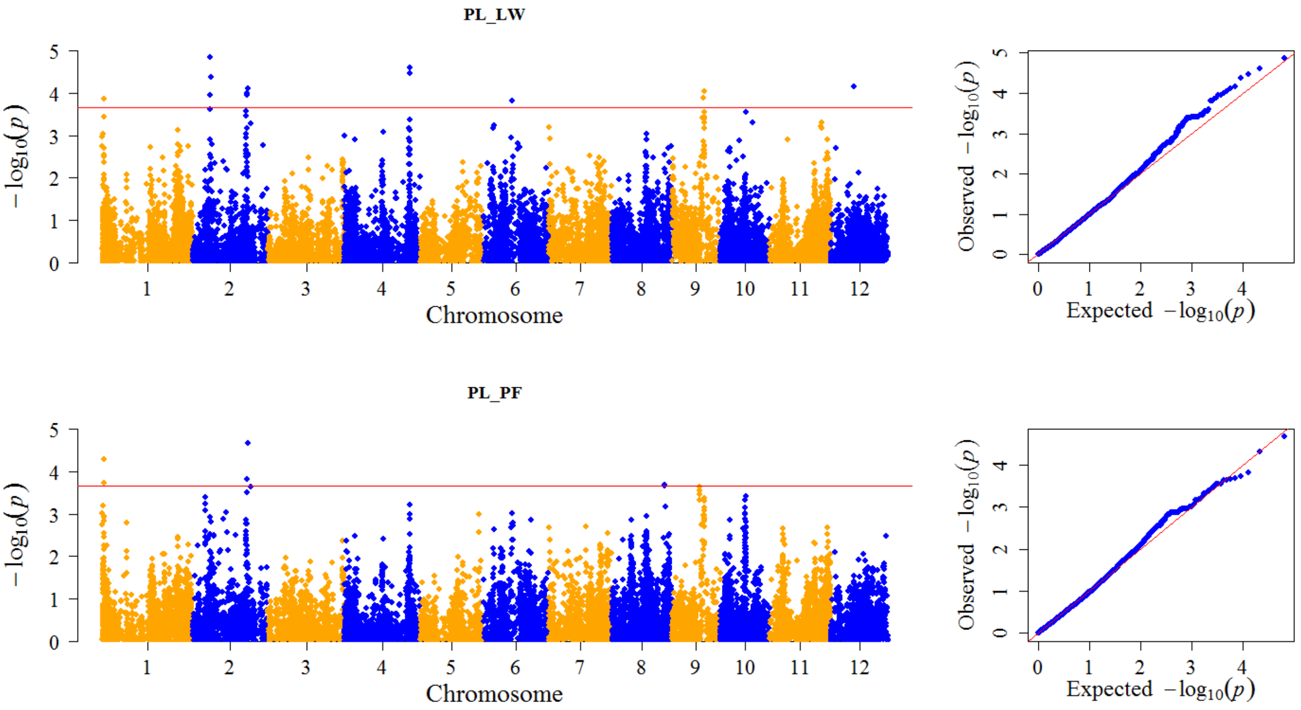

PL_LW
$-\log_{10}(p)$
Chromosome
1 2 3 4 5 6 7 8 9 10 11 12
Observed $-\log_{10}(p)$
Expected $-\log_{10}(p)$
PL_PF
$-\log_{10}(p)$
Chromosome
1 2 3 4 5 6 7 8 9 10 11 12
Observed $-\log_{10}(p)$
Expected $-\log_{10}(p)$

SEED MORPHOLOGY TRAITS

NSWLR_LW

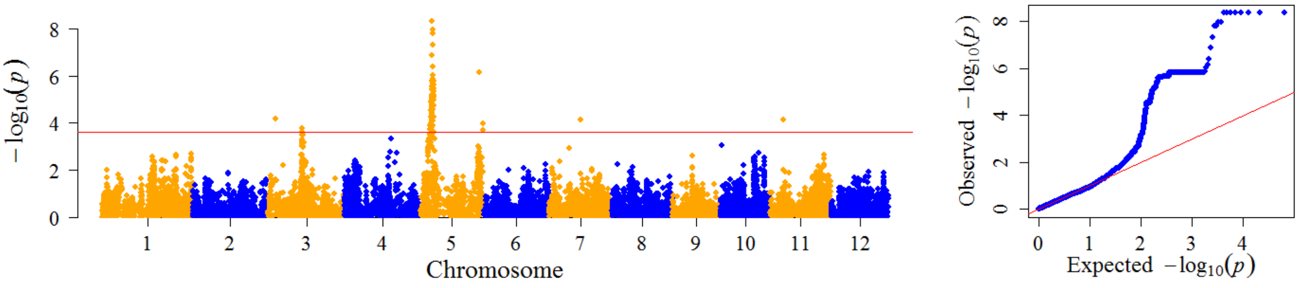

NSWLR_PF

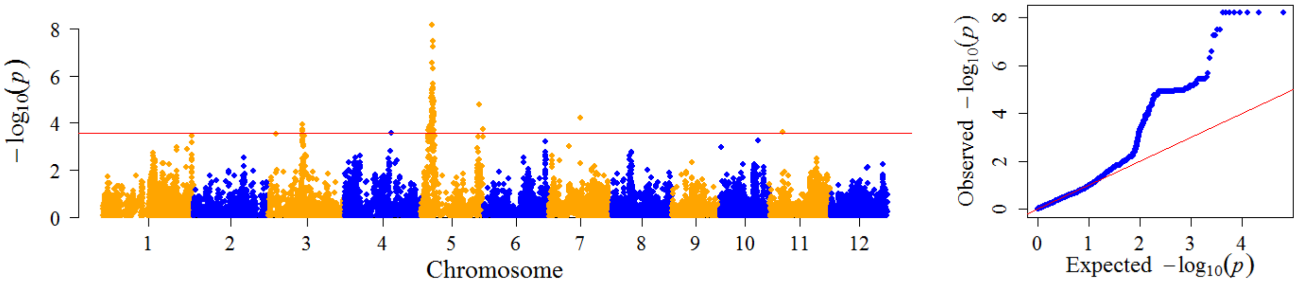

SWLR_LW

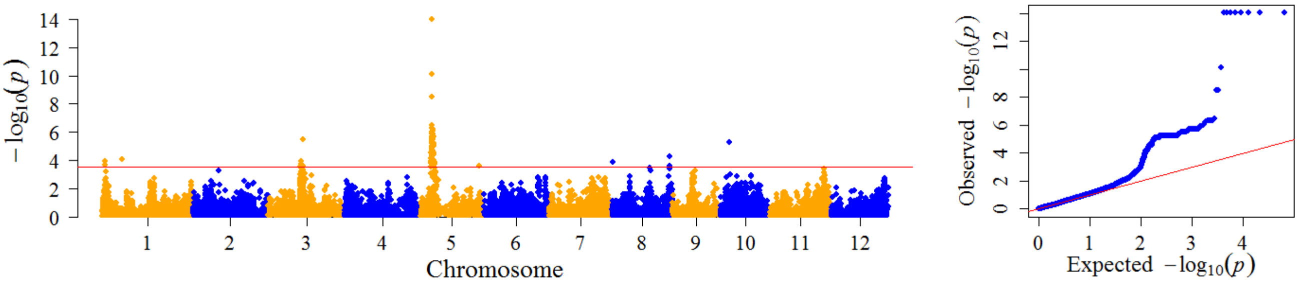

SWLR_PF

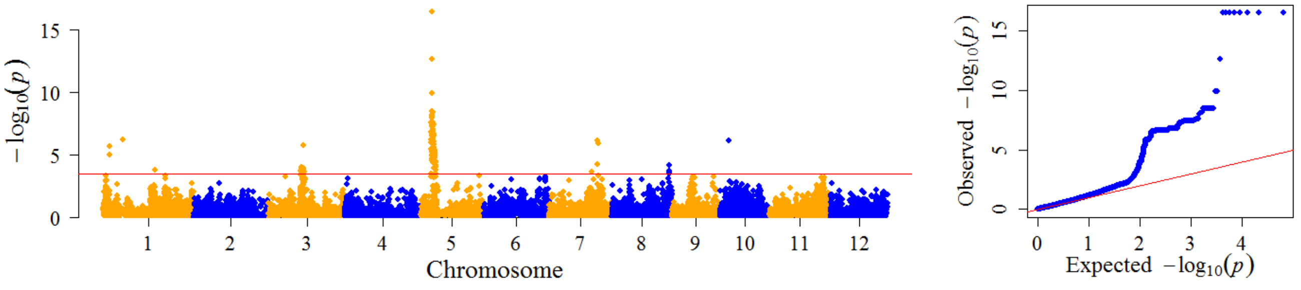

NSL_LW

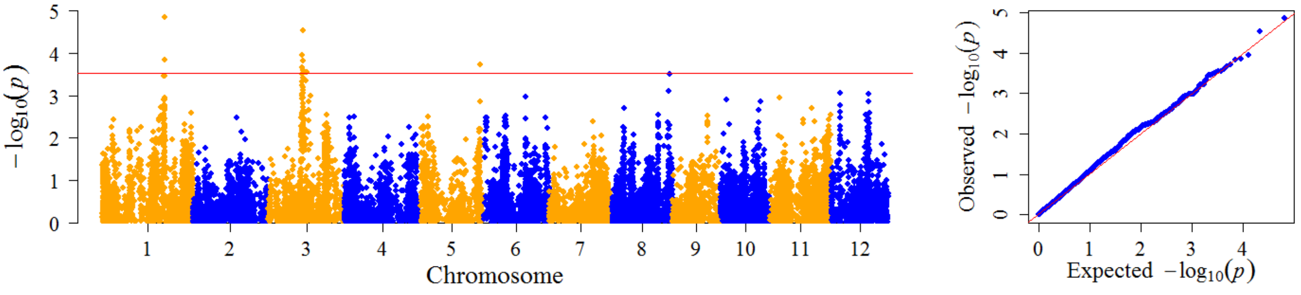

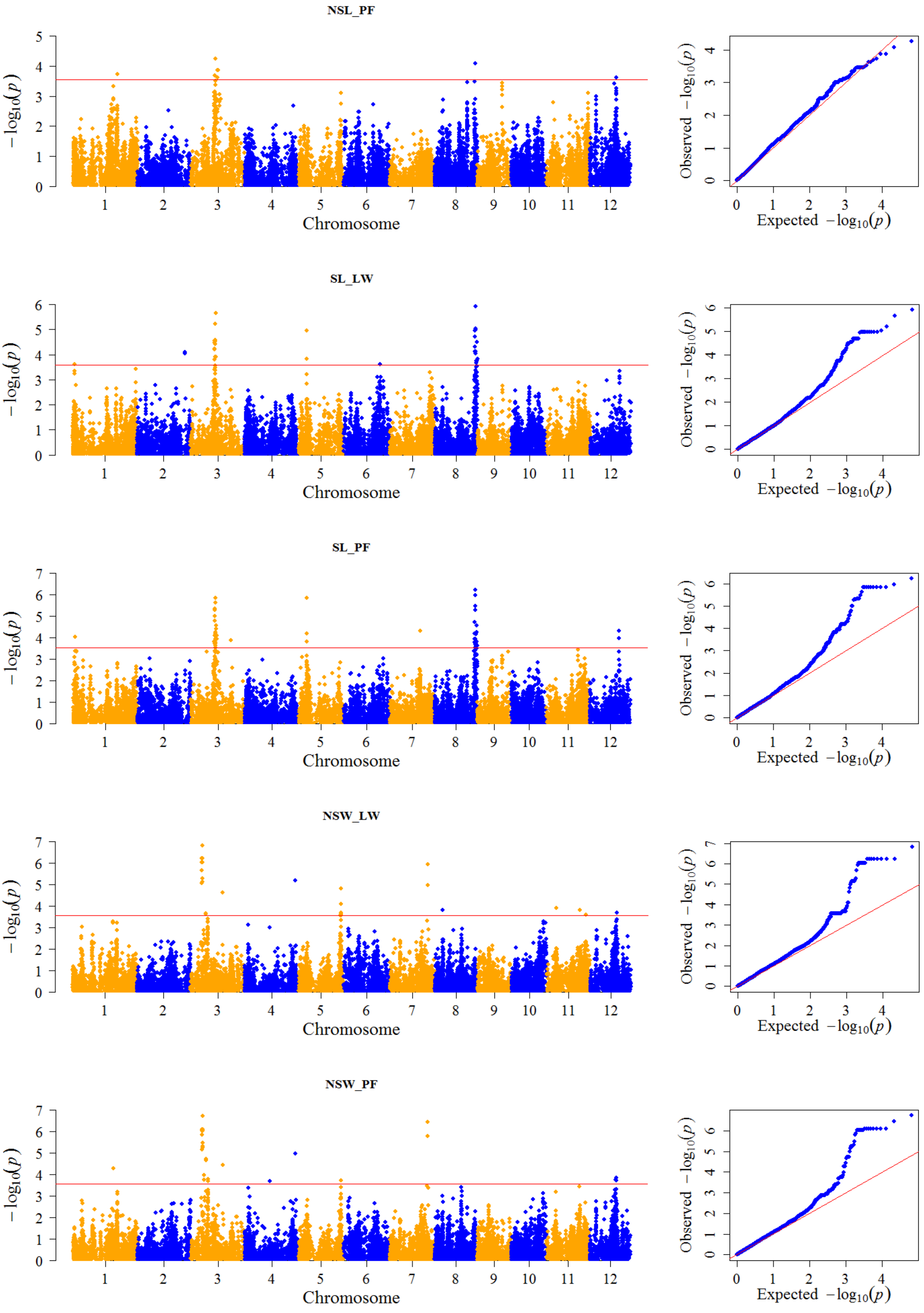
NSL_PF
SL_LW
SL_PF
NSW_LW
NSW_PF
$-\log_{10}(p)$
Chromosome
Observed $-\log_{10}(p)$
Expected $-\log_{10}(p)$

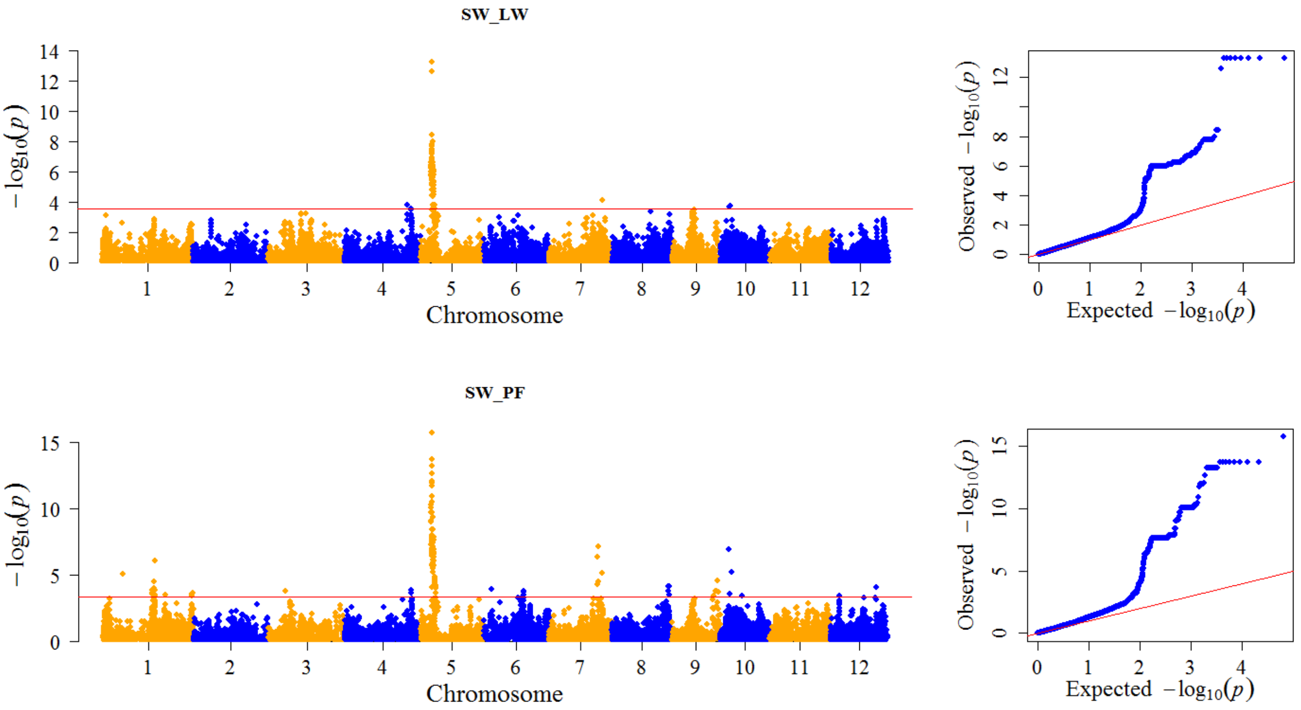
SW_LW
−log10(p)
Chromosome
Observed −log10(p)
Expected −log10(p)
SW_PF
−log10(p)
Chromosome
Observed −log10(p)
Expected −log10(p)

YIELD TRAITS

PW_LW

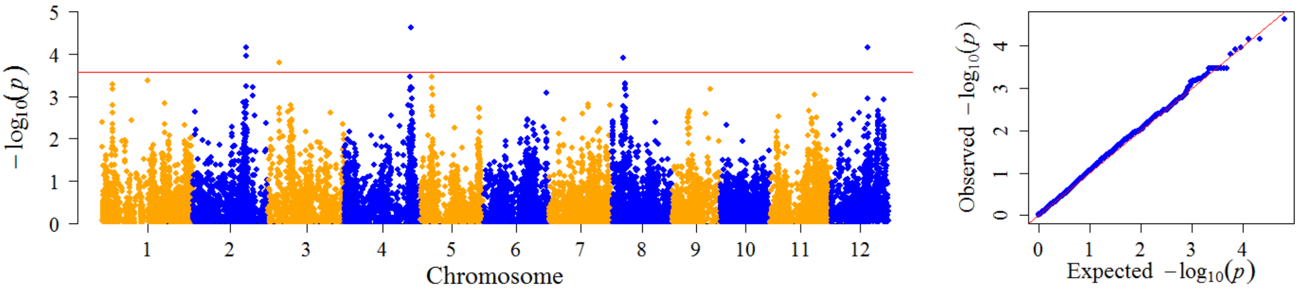

PW_PF

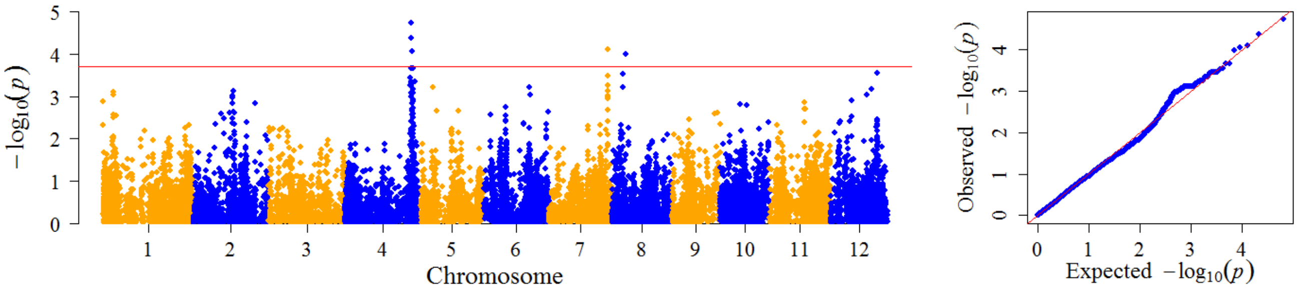

NHGW_LW

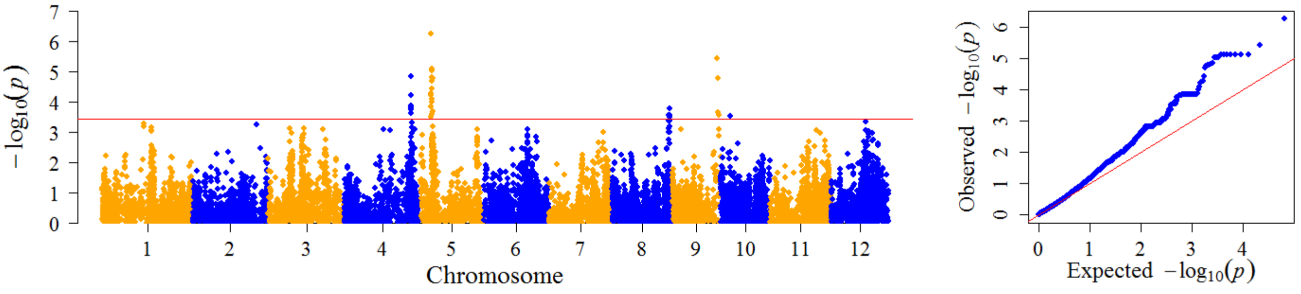

NHGW_PF

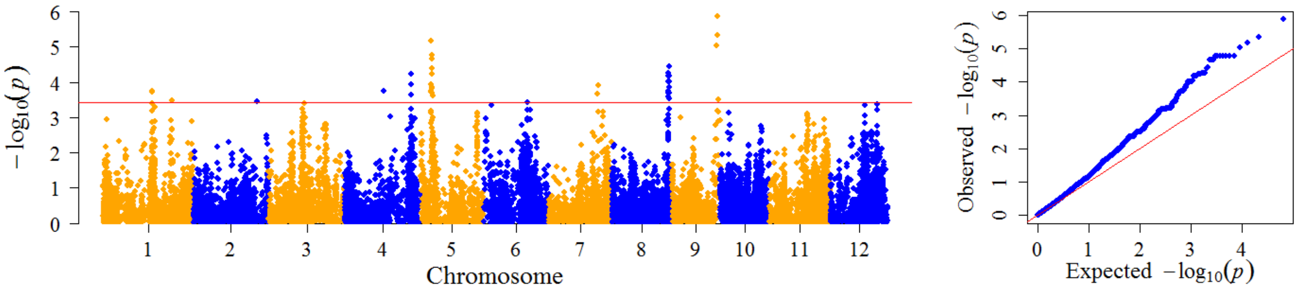

HGW_LW

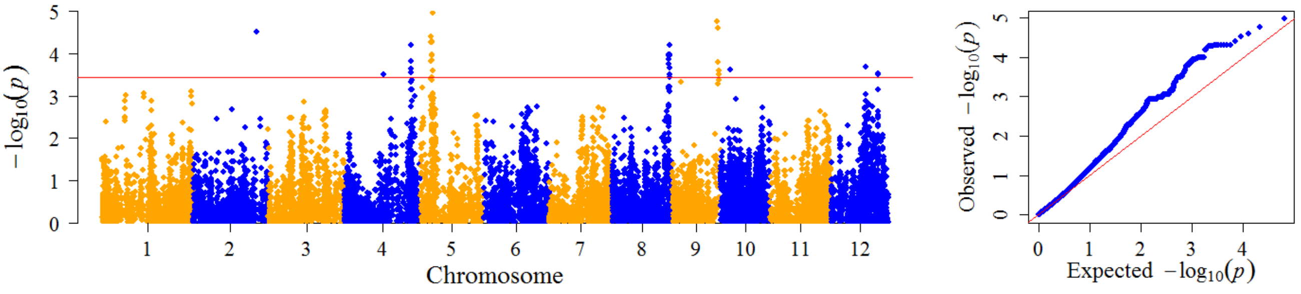

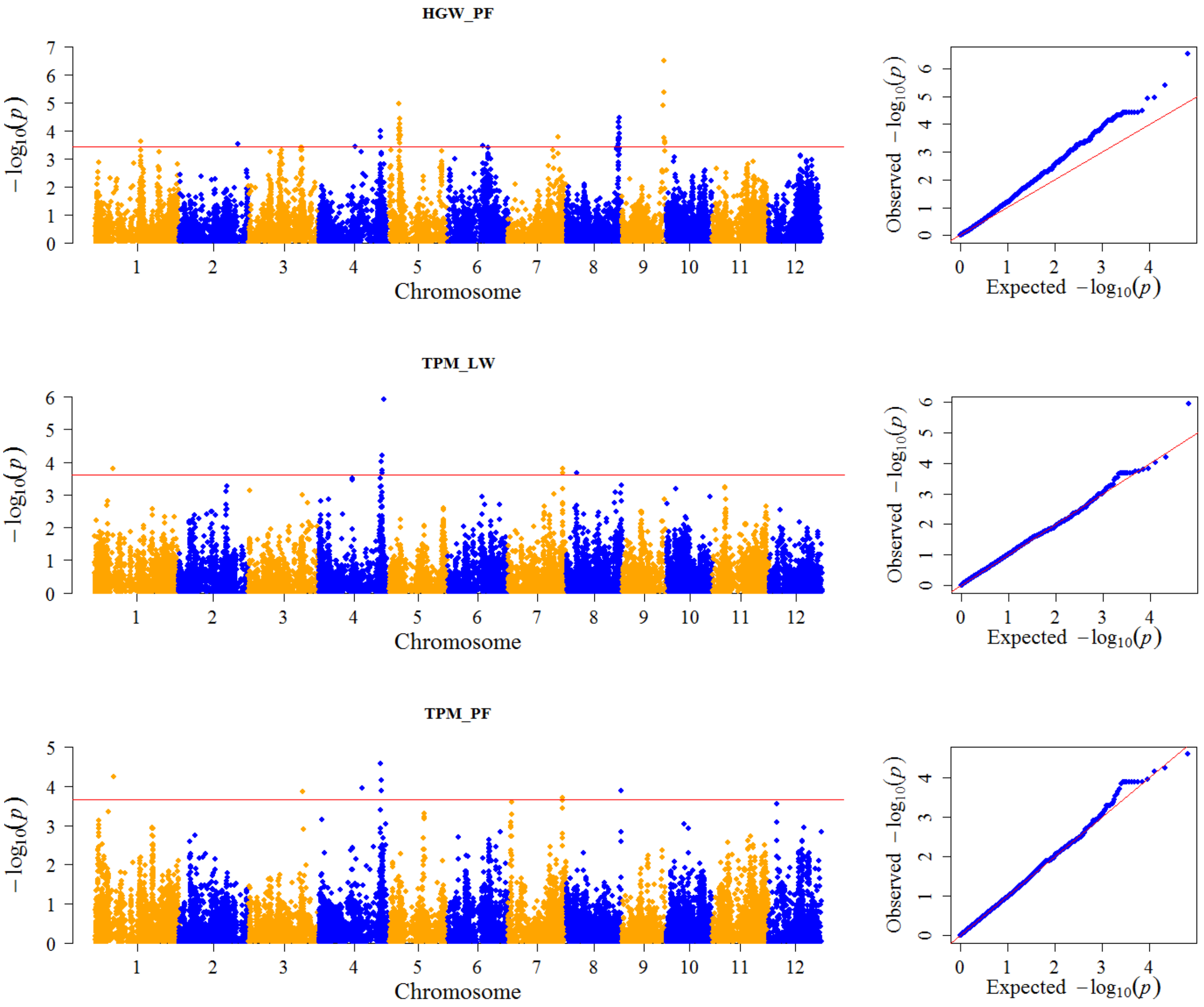
HGW_PF
TPM_LW
TPM_PF
$-\log_{10}(p)$
Chromosome
1 2 3 4 5 6 7 8 9 10 11 12
Observed $-\log_{10}(p)$
Expected $-\log_{10}(p)$

PHYSIOLOGY TRAITS

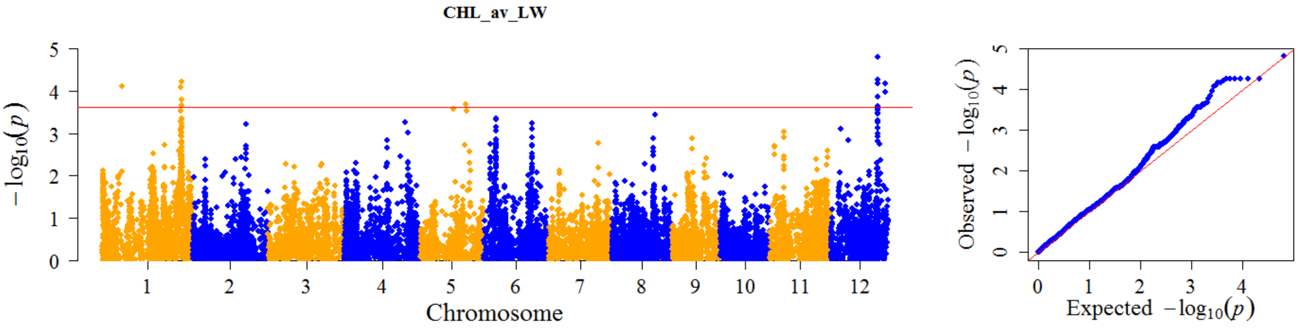

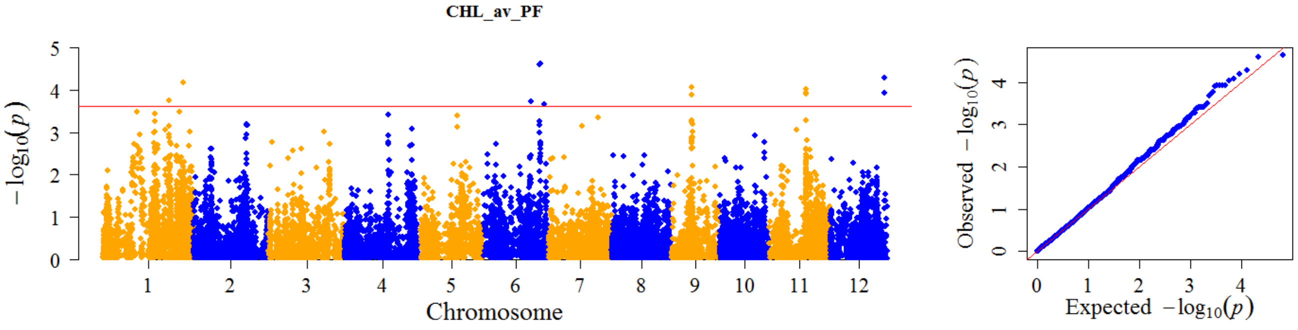

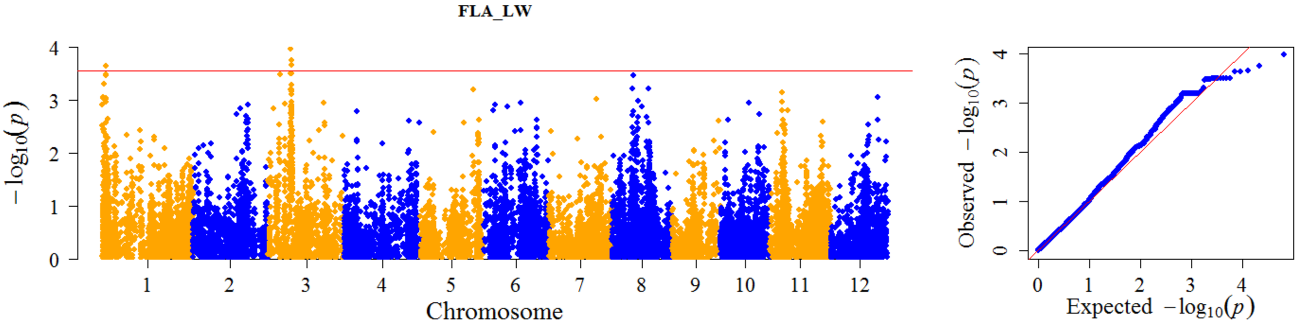

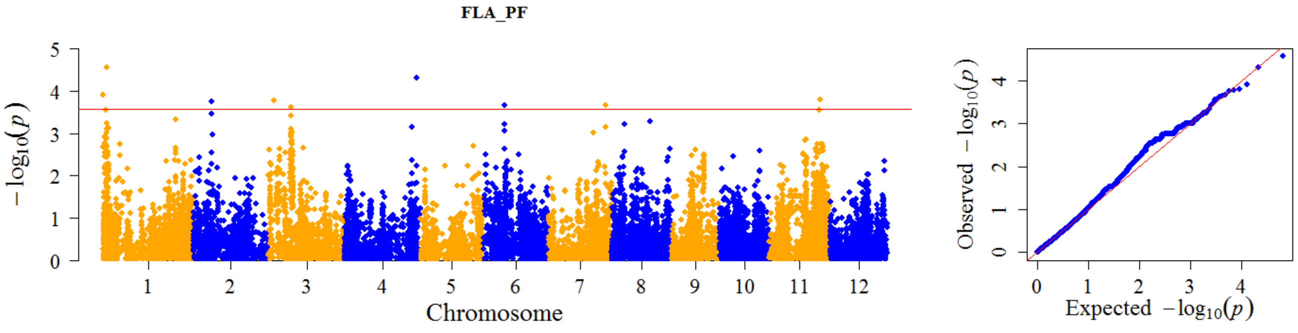

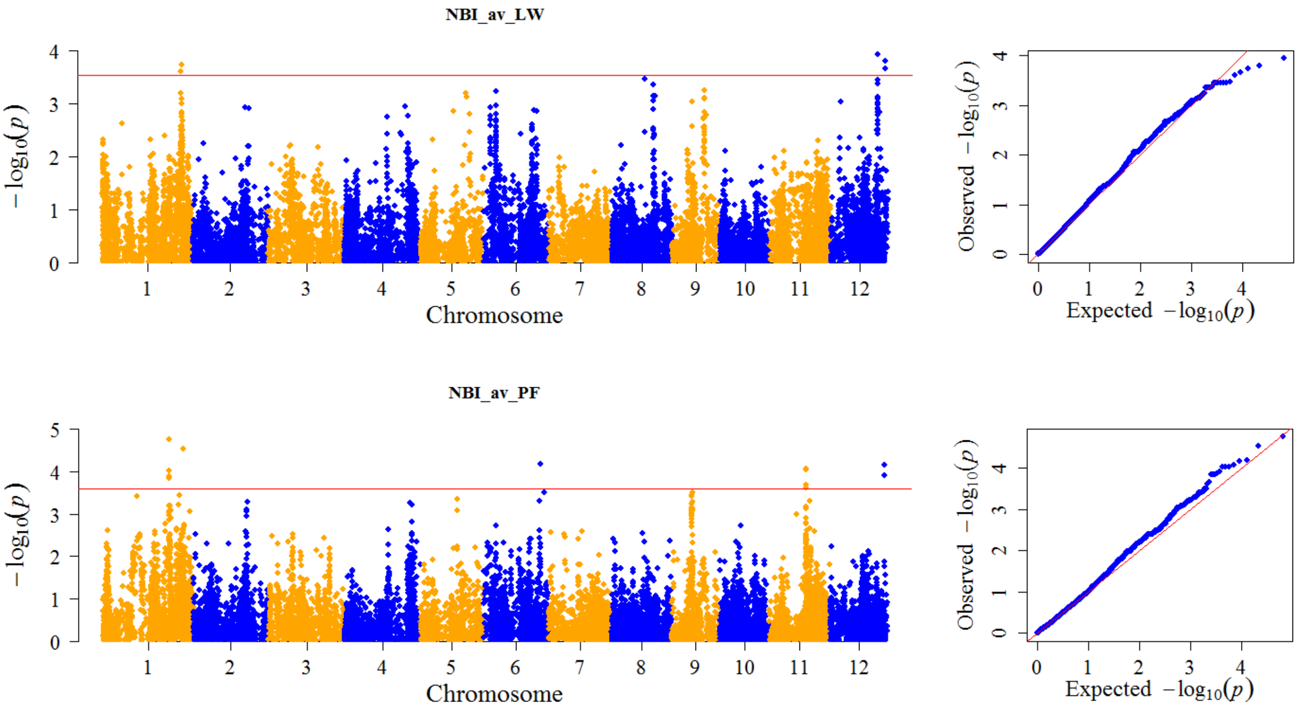
NBI_av_LW
$-\log_{10}(p)$
Chromosome
1 2 3 4 5 6 7 8 9 10 11 12
Observed $-\log_{10}(p)$
Expected $-\log_{10}(p)$
NBI_av_PF
$-\log_{10}(p)$
Chromosome
1 2 3 4 5 6 7 8 9 10 11 12
Observed $-\log_{10}(p)$
Expected $-\log_{10}(p)$
